# Functional mechanisms underlie the emergence of a diverse range of plasticity phenomena

**DOI:** 10.1371/journal.pcbi.1006590

**Published:** 2018-11-12

**Authors:** James A. Henderson, Pulin Gong

**Affiliations:** 1 School of Physics, The University of Sydney, Sydney, NSW, Australia; 2 ARC Centre of Excellence for Integrative Brain Function, The University of Sydney, Sydney, NSW, Australia; Research Center Jülich, GERMANY

## Abstract

Diverse plasticity mechanisms are orchestrated to shape the spatiotemporal dynamics underlying brain functions. However, why these plasticity rules emerge and how their dynamics interact with neural activity to give rise to complex neural circuit dynamics remains largely unknown. Here we show that both Hebbian and homeostatic plasticity rules emerge from a functional perspective of neuronal dynamics whereby each neuron learns to encode its own activity in the population activity, so that the activity of the presynaptic neuron can be decoded from the activity of its postsynaptic neurons. We explain how a range of experimentally observed plasticity phenomena with widely separated time scales emerge from learning this encoding function, including STDP and its frequency dependence, and metaplasticity. We show that when implemented in neural circuits, these plasticity rules naturally give rise to essential neural response properties, including variable neural dynamics with balanced excitation and inhibition, and approximately log-normal distributions of synaptic strengths, while simultaneously encoding a complex real-world visual stimulus. These findings establish a novel function-based account of diverse plasticity mechanisms, providing a unifying framework relating plasticity, dynamics and neural computation.

## Author summary

Many experiments have documented a variety of ways in which the connectivity strengths between neurons change in response to the activity of neurons. These changes are an important part of learning. However, it is not understood how such a diverse range of observations can be understood as consequences of an underlying algorithm used by brains for learning. In order to understand such a learning algorithm it is also necessary to understand the neural computation that is being learned, that is, how the functions of the brain are encoded in the activity of its neurons and its connectivity. In this work we propose a simple way in which information can be encoded and decoded in a network of neurons for operating on real-world stimuli, and how this can be learned using two fundamental plasticity rules that change the strength of connections between neurons in response to neural activity. Surprisingly, many experimental observations result as consequences of this approach, indicating that studying the learning of function provides a novel framework for unifying plasticity, dynamics, and neural computation.

## Introduction

Synaptic plasticity mechanisms shape the spatiotemporal dynamics of neural circuits that implement brain functions [1–4]. These plasticity mechanisms are usually divided into Hebbian and non-Hebbian mechanisms, where Hebbian changes in synaptic strength depend on both presynaptic and postsynaptic activity, but non-Hebbian mechanisms do not [5]. Among the most studied details of plasticity are its temporal characteristics, including dependencies on precise timings of neuronal activities in spike timing dependent plasticity (STDP), for example [6, 7]. Plasticity mechanisms operate over a diverse range of timescales, where experimentally Hebbian plasticity mechanisms are typically observed to be fast, and non-Hebbian, homeostatic mechanisms are much slower [5, 8, 9]. This is despite theoretical arguments suggesting fast acting homeostatic mechanisms are required to prevent runaway changes to synaptic strengths which would lead to either very high firing rates, or all activity dying out [8, 10, 11]. Further complicating this are observations of metaplasticity, whereby plasticity is modulated by past activity [12–14].

The interplay of this diverse range of plasticity mechanisms with network dynamics gives rise to complex spatial and temporal neuronal activity in which neurons fire in a very irregular way with variable spike timing and fluctuating firing rates [8, 15–18]. Experiments indicate that heterogeneous, approximately log-normal distributions of synaptic strengths form the network of physical connections underlying these dynamics [19, 20]. Often this distribution of connection strengths and spiking activity is arranged in a tight balance whereby excitatory inputs are closely tracked by inhibitory ones [21–23]. However, the fundamental questions of why such diverse plasticity phenomena emerge, and how their dynamics interact with neural circuits to give rise to these complex neural dynamics remain unclear [3, 5, 22, 24].

Some studies have developed models of plasticity by fitting to these detailed experimental results, for example the spike triplet based models [25, 26]. However, these types of models do not explain why the observed properties of plasticity are required for brain function by deriving them from more general functional principles. Further, these plasticity models are often combined ad-hoc to tune the dynamics in neural circuits. The resulting dynamics are often interpreted as having a potential functional role, for example in memory or decision making, but lack a convincing theoretical basis that would be provided by a deeper connection between the plasticity rules and more general functional principles [23, 26–30]. To deepen our understanding of synaptic plasticity it is important to unravel the links between these mechanisms and account for them in a unified way.

Plasticity mechanisms give neural circuits the ability to reorganise themselves for learning. In theoretical models and applications, effective learning in neural networks requires that plasticity mechanisms are carefully orchestrated by an overarching learning algorithm that is linked to performing a required task [31]. As whole entities, brains perform unsupervised learning in the sense that they do not learn with the aid of an external agent directly supervising the activity of certain target neurons. Instead, brains must learn to produce useful outputs using information obtained from sensory input. Understanding how to perform useful unsupervised learning is an important problem that is subject to ongoing investigation [32]. In machine learning, autoencoders perform unsupervised learning by learning an encoder that maps an input to a representation encoded in a layer of neurons, and a decoder that reconstructs the input from these neurons [31]. However, the usefulness of an autoencoder is not in performing this reconstruction, rather, it is the ability to extract useful features of the input for performing other tasks.

Here we show that diverse plasticity phenomena can emerge from a fundamental perspective of learning the function of a neural circuit. Specifically, we consider unsupervised learning in which each neuron learns to encode its own activity in the population activity so that the activity of the presynaptic neuron can be decoded from the activity of the postsynaptic neurons. In doing so, we extend the concept of an autoencoder in machine learning to recurrent networks [31]. Both Hebbian and non-Hebbian plasticity rules are required to account for the spiking statistics underlying this function and we derive a precise relationship between these rules.

We then demonstrate that a great variety of experimental observations emerge from this function based model. For synaptic plasticity mechanisms, the model contains the classic exponentially decaying timing dependence of STDP, as well as the frequency dependence of both STDP [33] and non-timing based stimulation experiments [34, 35]. The derived relation between Hebbian and non-Hebbian plasticity can be interpreted as a form of heterosynaptic metaplasticity. This relationship fluctuates over a range of timescales and resolves the paradox in which only slow homeostatic plasticity has been observed experimentally, but fast acting homeostatic plasticity is required by theoretical arguments to stabilise fast, unstable Hebbian plasticity [10, 11]. Our plasticity rules predict that classical STDP is a special case of more fundamental plasticity rules, and predict a spike timing dependence of non-Hebbian plasticity.

We further illustrate that when our plasticity rules are incorporated into a biologically realistic neural circuit of spiking neurons that are stimulated by a real-world visual stimulus, balanced excitation and inhibition is self organised and related to the stability of the neural dynamics. We show that approximately log-normal distributions of synaptic strengths emerge and are organised into receptive fields, all while the circuit learns to encode the complex real-world visual stimulus. These results thus present a novel, unified account of plasticity, dynamics and neural computation.

## Results

### A functional relationship between Hebbian and homeostatic rules

Typical approaches to learning in artificial neural networks define a global error function for a chosen set of ‘output’ neurons and compute a gradient of this error with respect to connection strengths for use as plasticity rules [31]. However, in the brain it is not clear what this set of output neurons might be and how they would all communicate their error globally [36].

**Fig 1:**
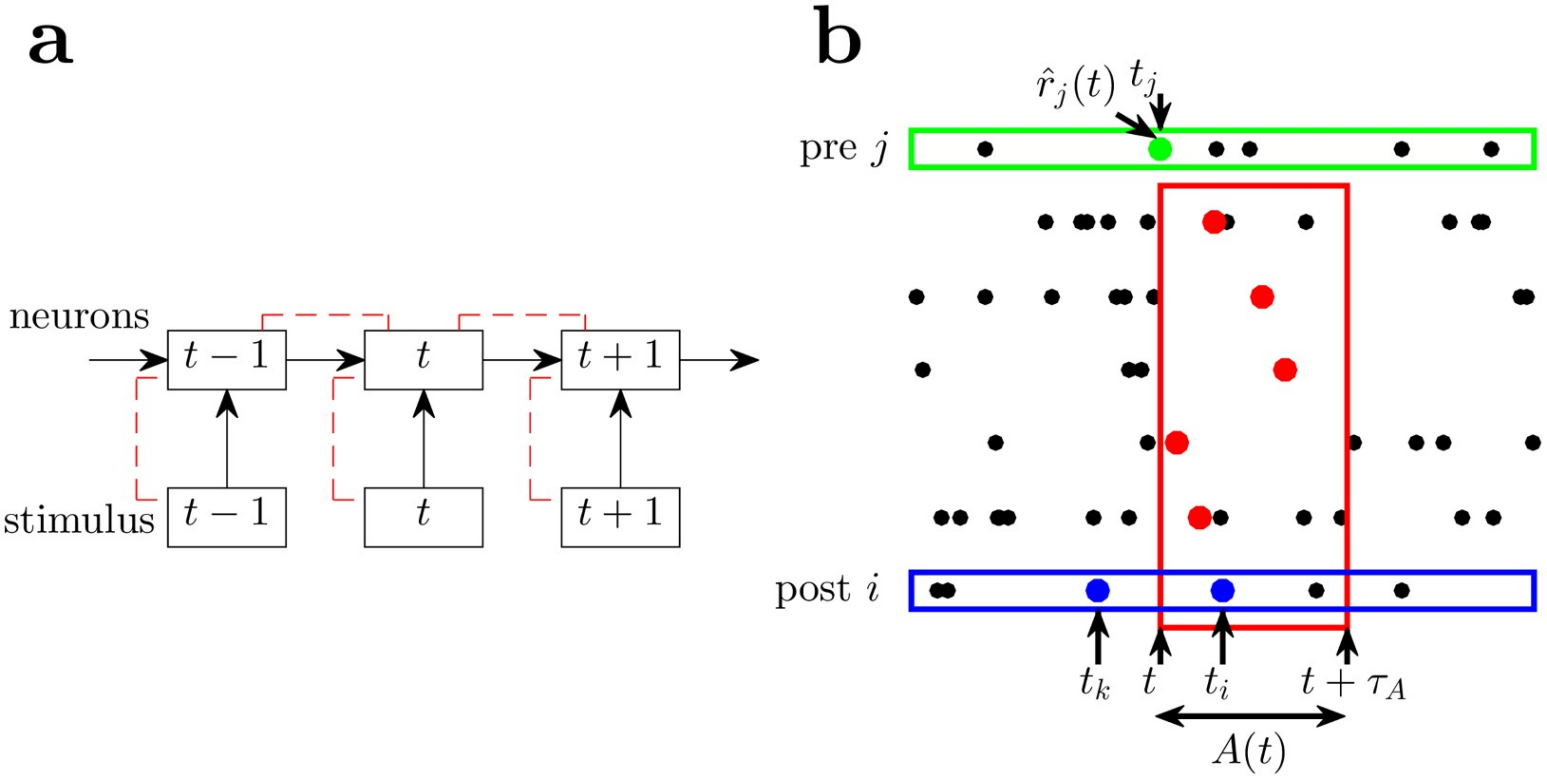
Learning and autoencoding framework for recurrent spiking networks. (a) We implement autoencoding in a recurrent network between the activity of the neurons across time, *t*. This can be visualised schematically where the activity of the network is unfolded in time, and the activity of the network at these timepoints become layers. Autoencoding between successive layers/timepoints is indicated by dotted red lines, with the same connections between each pair of layers/timepoints. Autoencoding also occurs in the traditional sense between the separate populations. (b) A schematic period of postsynaptic spiking activity *A*(*t*) enclosed in the red rectangle from time *t* to *t* + *τ*_*A*_ is used to compute the expected instantaneous firing rate ${\hat{r}}_{j}\left(t\right)$ of presynaptic neuron *j* (green) at time *t*. In this example, presynaptic neuron *j* spikes at *t* (but this does not always occur) so the Hebbian plasticity rule, Δ*w*^*H*^, is applied once to each synapse from presynaptic neuron *j* to the postsynaptic neurons that subsequently spike between *t* and *t* + *τ*_*A*_, for each postsynaptic neuron’s first spike only (red dots). In applying the non-Hebbian plasticity rule, Δ*w*^*nH*^, in response to a spike in postsynaptic neuron *i* (blue) at *t*_*i*_, the integration in Eq (6) is over the time period from the most recent previous spike *t*_*k*_ to the current spike at *t*_*i*_.

Instead, we begin by considering the function of neural circuits, focussing on local properties of individual neurons and their spiking activity using the concept of an autoencoder in machine learning [31]. In classical autoencoders, connections only exist between layers. However, neural circuits in brains are highly recurrent, so we consider autoencoding in a recurrent network where the activity of neurons is encoded and decoded across time, instead of distinct layers. This is a continuous process in time but can be visualised discretely by unfolding the recurrent network in time so that each ‘layer’ is the state of the neurons at successive timepoints, as in Fig 1a. We use tied weights meaning that encoding and decoding use the same set of connections.

Specifically, each neuron learns to encode and decode its own activity into and from a period of the population activity of its postsynaptic neurons, *A*(*t*), as in Fig 1b. The encoding of presynaptic neuron *j*’s spikes into postsynaptic activity, *A*(*t*), is familiar and is described by the neuronal and synaptic dynamics. The non-obvious part is how to describe the decoding of presynaptic neuron *j*’s activity from its postsynaptic activity, *A*(*t*), and ensuring that it approximates the actual activity of neuron *j*.

We define ${\hat{r}}_{j}\left(A\left(t\right)\right)$ to be the decoded estimate of presynaptic neuron *j*’s actual instantaneous firing rate, *r*_*j*_(*A*(*t*)), from the postsynaptic activity *A*(*t*). In a network operating in discrete Δ*t* timesteps (as is the case for simulations) we can relate this to the conditional probability of presynaptic neuron *j* spiking in conjunction with the pattern of postsynaptic activity *A* that happens to occur during the time window from *t* to *t* + *τ*_*A*_, that is $\Delta tr_{j}\left(A\right)=p\left(j\;\text{spiking}|A\right)$. We want learning to ensure that $\Delta t{\hat{r}}_{j}\left(A\right)\approx p\left(j\;\text{spiking}|A\right)$.

Although we have not yet defined the decoded instantaneous presynaptic firing rate, ${\hat{r}}_{j}\left(A\left(t\right)\right)$, it should be encoded in the network using local properties of the network that neuron *j* has access to, so that neuron *j* has the ability to directly encode and decode its own activity. The purpose of learning is to modify the network so that the encoding and decoding processes are able to (approximately) invert each other, since in any arbitrary configuration the decoding of presynaptic neuron *j*’s activity from its postsynaptic activity, *A*(*t*), will likely be poor.

We continue by applying learning rules directly to the decoded instantaneous presynaptic firing rate, ${\hat{r}}_{j}\left(A\left(t\right)\right)$, not the properties of the network used to encode it. Later, we specify how the decoded instantaneous presynaptic firing rate, ${\hat{r}}_{j}\left(A\left(t\right)\right)$, is decoded from the connectivity, *W*, and the postsynaptic activity *A*(*t*). We use this to translate the learning rules that operate directly on the decoded instantaneous presynaptic firing rate, ${\hat{r}}_{j}\left(A\left(t\right)\right)$, into plasticity rules for modifying connection strengths, *W*, and demonstrate learning in a spiking neural circuit with a real-world, complex visual stimulus. We do not model the detailed physiology of the synapses represented by *W* and the mechanisms by which they change, but it should be noted that the resulting neural dynamics can depend on these lower level details, for example, if synaptic changes are presynaptically or postsynaptically expressed [37].

Given a long period of activity in a network, there are two events that need to be counted to estimate the instantaneous presynaptic firing rate, *r*_*j*_(*A*(*t*)). Firstly, the number of times that postsynaptic activity, *A*(*t*), occurs, and secondly, the number of times that presynaptic neuron *j* spikes (within an arbitrarily short Δ*t*) in conjunction with postsynaptic activity, *A*(*t*). This second event is a Hebbian event as it consists of both postsynaptic activity, *A*(*t*), and a presynaptic *j* spike, while the first event is non-Hebbian since it only consists of postsynaptic activity, *A*(*t*) [4, 5]. Thus, both Hebbian and non-Hebbian learning are required to account for the statistics of the learned activity.

We apply Hebbian, *R*_*H*_, and non-Hebbian, *R*_*nH*_, learning rules to the decoded instantaneous presynaptic firing rate, ${\hat{r}}_{j}\left(A\left(t\right)\right)$, in response to each of these events to iteratively learn so that eventually the decoded instantaneous presynaptic firing rate, ${\hat{r}}_{j}\left(A\left(t\right)\right)$, converges to the actual instantaneous presynaptic firing rate, *r*_*j*_(*A*(*t*)). After a time period in which a particular pattern of postsynaptic activity, *A*(*t*), occurs *N* times, a series of iterated operations of the non-Hebbian, *R*_*nH*_, and Hebbian, *R*_*H*_, rules are applied to the initial value of the decoded instantaneous presynaptic firing rate, ${\hat{r}}_{j}^{0}\left(A\left(t\right)\right)$;

$$\begin{array}{c} {\hat{r}}_{j}^{N}\left(A\left(t\right)\right)=(R_{nH}\circ R_{H})\circ ...\circ (R_{nH}\circ R_{H})\circ {\hat{r}}_{j}^{0}\left(A\left(t\right)\right), \end{array}$$ (1)

where ∘ denotes functional composition, and brackets group operations for each occurrence of the postsynaptic activity *A*(*t*), noting that the Hebbian rule, *R*_*H*_, is absent if presynaptic neuron *j* does not spike coincident with postsynaptic activity, *A*(*t*).

From the decoding target ${\hat{r}}_{j}^{N}\left(A\left(t\right)\right)=r_{j}\left(A\left(t\right)\right)$, we derive a fundamental relationship between the Hebbian and non-Hebbian learning rules;

$$\begin{array}{c} \Delta R_{nH}\left({\hat{r}}_{j}\right)=-\Delta t{\hat{r}}_{j}\Delta R_{H}\left({\hat{r}}_{j}\right), \end{array}$$ (2)

where Δ*R*_*nH*_ and Δ*R*_*H*_ are the changes to $\hat{r}$ due to each application of the non-Hebbian, *R*_*nH*_, and Hebbian, *R*_*H*_, learning rules, respectively. This relationship describes how the Hebbian and non-Hebbian changes should be related so that they cancel each other and no change occurs when $\hat{r}\left(A\left(t\right)\right)$, the estimate of the instantaneous presynaptic firing rate given some postsynaptic activity *A*(*t*), equals *r*(*A*(*t*)), the actual presynaptic firing rate given *A*(*t*).

To ensure convergence, the non-Hebbian, *R*_*nH*_, and Hebbian, *R*_*H*_, rules must slightly decrease and increase the decoded instantaneous presynaptic firing rate, ${\hat{r}}_{j}$, respectively (for further details of the derivation see S1 Appendix). This relationship between the learning rules matches the statistical relationship between the Hebbian and non-Hebbian events, remembering that $\Delta tr_{j}\left(A\right)=p\left(j\;\text{spiking}|A\right)$, whereby typically Hebbian events must make larger changes than non-Hebbian events because they occur less frequently.

### Plasticity from function

**Fig 2:**
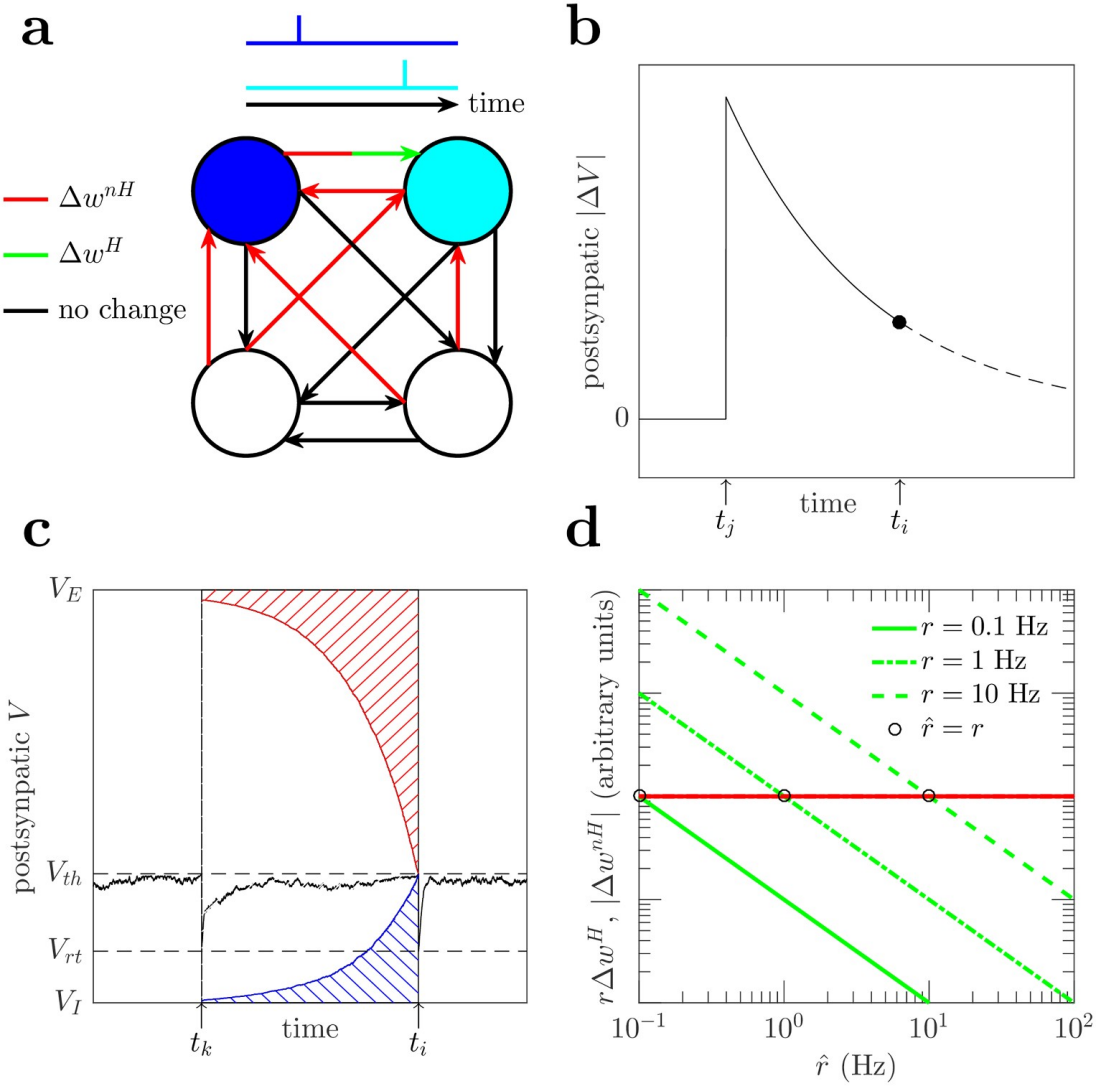
Plasticity rules. (a) Schematic of weight changes in response to a pair of spikes. Red and green indicate the applications of the non-Hebbian and Hebbian plasticity rules respectively. When the blue and cyan neurons spike, all their inputs are reduced via the non-Hebbian plasticity rule, Δ*w*^*nH*^. When the cyan neuron spikes, its connection from the blue neuron (red/green arrow) is increased via the Hebbian plasticity rule, Δ*w*^*H*^. Inputs to non-spiking neurons (black arrows) are unchanged. (b) The postsynaptic membrane potential change (black curve) from the presynaptic *j* spike decays until the time of the postsynaptic *i* spike (black dot). This corresponds to the (*V*_*i*_(*t*_*j*_) − *V*_*E*/*I*_)*e*^−Δ*t*/*τ*^ terms in $\hat{r}$ and Δ*w*^*H*^ in Eqs (3) and (4). (c) A schematic of the non-Hebbian plasticity rule (Eq (6)). The exponentially decayed difference between the postsynaptic membrane potential, *V*_*i*_, (black curve) and the synaptic reversal potential, *V*_*E*/*I*_, is integrated over the time period between postsynaptic spikes at *t*_*k*_ and *t*_*i*_. Red and blue hashing are the integral for excitatory and inhibitory synapses, respectively. (d) For a given pattern of postsynaptic activity, *A*(*t*), the total expected Hebbian weight change, *r*Δ*w*^*H*^, (green lines) varies with the decoded presynaptic instantaneous firing rate, $\hat{r}$, but the non-Hebbian weight change, Δ*w*^*nH*^, (red line) does not. When $\hat{r}>r$, then the non-Hebbian weight change is larger than the Hebbian weight change, so the connection strength, *w* and $\hat{r}$ decrease, and vice versa when $\hat{r}<r$. The intersections of the red and green lines occur when $\hat{r}=r$ (black circles) and the Hebbian and non-Hebbian weight changes cancel each other. Hebbian weight changes are plotted for three different presynaptic firing rates (0.1, 1, 10 Hz).

Now that we have established a relationship between Hebbian and non-Hebbian learning rules acting on the decoded instantaneous presynaptic firing rate, ${\hat{r}}_{j}$, we consider how these learning rules may be implemented in a neural circuit as plasticity rules acting on synapses (Fig 2a).

In recurrent networks it is necessary to relate learning to the stability of the network to ensure that the network is stable [38]. Indeed, non-Hebbian, homeostatic plasticity is often invoked in models for the purpose of stabilizing unstable Hebbian plasticity. To control the stability of learning in a more principled way, we choose the decoded instantaneous presynaptic firing rate, ${\hat{r}}_{j}\left(A\left(t\right)\right)$, to be such that when presynaptic neuron *j* spikes at *t*, ${\hat{r}}_{j}\left(A\left(t\right)\right)$ is proportional to presynaptic neuron *j*’s contribution to generating its postsynaptic spikes, as shown schematically in Fig 1b. Thus learning the decoded instantaneous presynaptic firing rate, ${\hat{r}}_{j}\left(A\left(t\right)\right)$, also controls the individual gains for each neuron in the network. Further, if the neurons are divided into different populations, then we can compute the decoded instantaneous presynaptic firing rate, ${\hat{r}}_{j}^{\alpha \beta }\left(t\right)$, for presynaptic neuron *j* in population *α* at time *t*, decoded from postsynaptic neurons in population *β*, and therefore control the gains of interactions between different populations of neurons.

The change in membrane potential of a postsynaptic neuron *i* caused by a presynaptic spike in neuron *j*, decayed according to the leaky dynamics of the postsynaptic neuron, is well approximated a short time after the presynaptic spike occurs by *w*_*ij*_(*V*_*i*_(*t*) − *V*_*E*/*I*_)*e*^−Δ*t*/*τ*^/*C*, where Δ*t* is the time since the presynaptic spike and *V*_*E*/*I*_ is the excitatory or inhibitory reversal potential of the synapse, *τ* is the decay constant of the postsynaptic neuron’s membrane potential, *w*_*ij*_ is the synaptic conductance from presynaptic neuron *j* to postsynaptic neuron *i*, and *C* is the capacitance of the postsynaptic neuron (see Fig 2b and Methods). Thus, the decoded instantaneous presynaptic firing rate, ${\hat{r}}_{j}^{\alpha \beta }\left(t\right)$, is given by summing the contributions from the first spike after *t* in the postsynaptic activity, *A*^*β*^(*t*), for each postsynaptic neuron in population *β*. Only the first spike is considered since the reset and refractory state mean the presynaptic spike does not contribute to further postsynaptic spikes (Fig 1b),

$$\begin{array}{c} {\hat{r}}_{j}^{\alpha \beta }\left(t\right)=\gamma^{\alpha \beta }\sum_{i\in A^{\beta }\left(t\right)}\frac{w_{ij}}{C}\left(V_{i}\left(t\right)-V_{E/I}\right)e^{-(t_{i}-t)/\tau }, \end{array}$$ (3)

where *t*_*i*_ is the time at which postsynaptic neuron *i* first spikes after time *t*, and *γ*^*αβ*^ is a constant that scales the summed voltage changes into an instantaneous firing rate. Its value will be described in Sec. Emergent stability and balanced excitation and inhibition. Note that the decoded instantaneous presynaptic firing rate, ${\hat{r}}_{j}^{\alpha \beta }\left(t\right)$, can be computed at any *t*, independent of the activity of presynaptic neuron *j*, and is a local quantity, only requiring information about postsynaptic neurons to which presynaptic neuron *j* has a direct connection. This defines the decoding component of the autoencoder, describing how presynaptic activity can be decoded from postsynaptic activity.

### Hebbian plasticity

Given the expression in Eq (3) for computing the decoded instantaneous presynaptic firing rate, ${\hat{r}}_{j}\left(t\right)$, we now construct plasticity rules that modify the connection strengths, *w*_*ij*_. The collective changes to the connection strengths from all applications of these plasticity rules implement the Hebbian, *R*_*H*_, and non-Hebbian, *R*_*nH*_, learning rule changes to the decoded instantaneous presynaptic firing rate, ${\hat{r}}_{j}\left(t\right)$. We distinguish Hebbian, *R*_*H*_, and non-Hebbian, *R*_*nH*_, *learning* rules from their constituent lower level Hebbian, Δ*w*^*H*^, and non-Hebbian, Δ*w*^*nH*^, *plasticity* rules which are comparable to plasticity experiments.

Firstly, when a presynaptic neuron *j* spikes at time *t* the Hebbian plasticity rule, $\Delta w_{ij}^{H}$, is applied to the corresponding weight for each postsynaptic neuron *i* that subsequently spikes within the time period *τ*_*A*_ that defines the temporal extent of the postsynaptic activity, *A*(*t*) (Fig 1b). This time period *τ*_*A*_ is chosen so that the presynaptic spike’s contribution to the postsynaptic neuron’s membrane potential and corresponding contribution to the decoded instantaneous presynaptic firing rate, ${\hat{r}}_{j}\left(t\right)$, has decayed to be negligible after a delay of *τ*_*A*_.

The weight change is chosen to depend on the postsynaptic neuron’s contribution to the decoded instantaneous presynaptic firing rate, ${\hat{r}}_{j}^{\alpha \beta }\left(t\right)$, in Eq (3), see Fig 2b. This choice of credit assignment strengthens synapses to postsynaptic neurons whose spike generation relies strongly on the presynaptic spike from *j*. This creates competition between postsynaptic neurons and thus diversification of neural responses, including the development of receptive fields (see Sec. Self-organization of a spatially extended spiking neural network with complex real-world stimulus) [39]. So,

$$\begin{array}{c} \Delta w_{ij}^{H}\left(t_{j}+\tau_{A}\right)=\mp \epsilon \frac{w_{ij}\left(V_{i}\left(t_{j}\right)-V_{E/I}\right)e^{-(t_{i}-t_{j})/\tau }}{C{\hat{r}}_{j}^{\alpha \beta }\left(t_{j}\right)}, \end{array}$$ (4)

where *ϵ* is a small learning rate. The negative and positive signs are for excitatory and inhibitory synapses respectively and ensure that the weight change is (usually) positive, thus implementing LTP.

The decoded instantaneous presynaptic firing rate, ${\hat{r}}_{j}\left(t\right)$, requires information about all of presynaptic neuron *j*’s postsynaptic neurons. We do not describe the biological mechanisms that might implement this. However, it seems plausible that this information would be available to *j* by integration of signals from its postsynaptic neurons; though this is a weaker locality than is often assumed, where locality is restricted to neurons on either end of a synapse [10].

The inclusion of the decoded instantaneous presynaptic firing rate, ${\hat{r}}_{j}^{\alpha \beta }$, in Eq (4) modulates the size of the Hebbian change in response to past activity and can be interpreted as a form of metaplasticity [12–14]. This is because the decoded instantaneous presynaptic firing rate, $\hat{r}$, depends both on the connection strengths which change slowly over time due to accumulated small plasticity changes in response to neural activity, and also the immediate postsynaptic activity which evolves rapidly in time.

Other plasticity rules can be constructed that together still obey the required relationship between the Hebbian, *R*^*H*^, and non-Hebbian, *R*^*nH*^ learning rules described in Eq (2). In particular one possibility is to include $\hat{r}$ in the non-Hebbian plasticity rule instead of the Hebbian plasticity rule. Implementing this version of the non-Hebbian plasticity rule in simulations would be computationally very expensive as it would require computing $\hat{r}$ at every timestep as part of the integral in Eq (6), rather than only at the time of presynaptic spikes when included in the Hebbian rule in Eq (4); however, this may not be a limitation in biology.

### Non-Hebbian plasticity

Based on the functional relationship between Hebbian and non-Hebbian rules in Eq (2), we obtain a non-Hebbian plasticity rule from Eq (4),

$$\begin{array}{c} \frac{d\Delta w_{ij}^{nH}\left(t\right)}{dt}=\pm \epsilon \frac{w_{ij}\left(V_{i}\left(t\right)-V_{E/I}\right)e^{-(t_{i}-t)/\tau }}{C}, \end{array}$$ (5)

where *t*_*k*_ < *t* < *t*_*i*_. We integrate this over the time period between subsequent postsynaptic *i* spikes to yield a discrete weight change applied in response to postsynaptic neuron *i* spiking;

$$\begin{array}{c} \Delta w_{ij}^{nH}\left(t_{i}\right)=\pm \epsilon \frac{w_{ij}}{C}\int_{t_{k}}^{t_{i}}\left(V_{i}\left(t^{\prime }\right)-V_{E/I}\right)e^{-(t_{i}-t^{\prime })/\tau }dt^{\prime }, \end{array}$$ (6)

where *t*_*k*_ is the time of the postsynaptic *i* spike before *t*_*i*_ (Fig 1b), and the positive and negative signs are for excitatory and inhibitory synapses respectively and ensure that the weight change is (usually) negative and implements LTD, as illustrated in Fig 2c.

This non-Hebbian plasticity rule can be considered a homeostatic mechanism that scales down synaptic strengths [4, 5, 23]. This plasticity rule is local, only using information about neurons on either end of a synapse, and predicts a form of non-Hebbian spike timing dependence, whereby the magnitude of the change depends exponentially on the timing between subsequent pairs of postsynaptic spikes.

In summary, the form of the combined change from both plasticity rules can be described qualitatively by a Hebbian term $(\epsilon /\hat{r})\times pre\times post$ and a non-Hebbian term *ϵ* × *post*,

$$\begin{array}{c} \Delta w∼\frac{\epsilon }{\hat{r}}(pre-\hat{r})post, \end{array}$$ (7)

where *pre* indicates that the Hebbian term requires a presynaptic spike, and *post* indicates the dependence on postsynaptic membrane potential and spiking as in Eqs (4) and (6). This equation has a stable fixed point at $\hat{r}=pre$. Thus, these two weight changes reach a balance where the size and frequency of each of the weight updates cancel each other on average over time so that the learned quantity, $\hat{r}$, corresponding to the decoded instantaneous presynaptic firing rate, approximates the actual presynaptic instantaneous firing rate, *r*, as shown in Fig 2d.

This weight change is similar to that presented in [23] where plasticity takes the form Δ*w* ∼ *ϵpre*(*post* − *ρ*_0_). In [23] the fixed point occurs when the postsynaptic activity reaches the fixed target firing rate *ρ*_0_, and so the activity of the network is directed toward this fixed firing rate.

The crucial differences to the plasticity rules presented here are that firstly, here the fixed point involves the presynaptic, not the postsynaptic activity. Secondly, the decoded instantaneous presynaptic firing rate, $\hat{r}$, varies and does not represent a fixed target firing rate like *ρ*_0_ as in [23]. Instead, the actual presynaptic firing rate *r* can take any value and is a target for the decoded instantaneous presynaptic firing rate, $\hat{r}$, which is modifiable and depends on the postsynaptic activity. Thus, neuronal firing rates can fluctuate over time in response to stimuli or other recurrent activity.

It is possible to use the same approach we have taken to develop plasticity rules that perform supervised learning in which input connections to a neuron are learned and thus directly control postsynaptic firing rates, with a variable target postsynaptic firing rate provided by an external agent. This supervised learning formulation leads to a weight change of the form $\Delta w∼\left(\epsilon /\hat{r}\right)pre(post-\hat{r})$. In this case, presynaptic and postsynaptic activity are swapped compared to Eq (7) and $\hat{r}$ becomes the target postsynaptic supervision signal, but the details of this are left for future work. With this in mind, we can then interpret the plasticity rules presented in [23] as implementing supervised learning, but with the postsynaptic supervision signal being a simple fixed firing rate, *ρ*_0_.

### Comparison to classical STDP

We next show that these plasticity rules (Eqs 4 and 6) reproduce the characteristics of a variety of experimentally observed plasticity phenomena [1, 7, 33–35, 40]. First we demonstrate these plasticity rules capture an exponential timing dependence in the classic model of STDP, derived from experiments in which pairs of presynaptic and postsynaptic spikes with time delay Δ*t* are repeated at a fixed frequency [6, 7].

**Fig 3:**
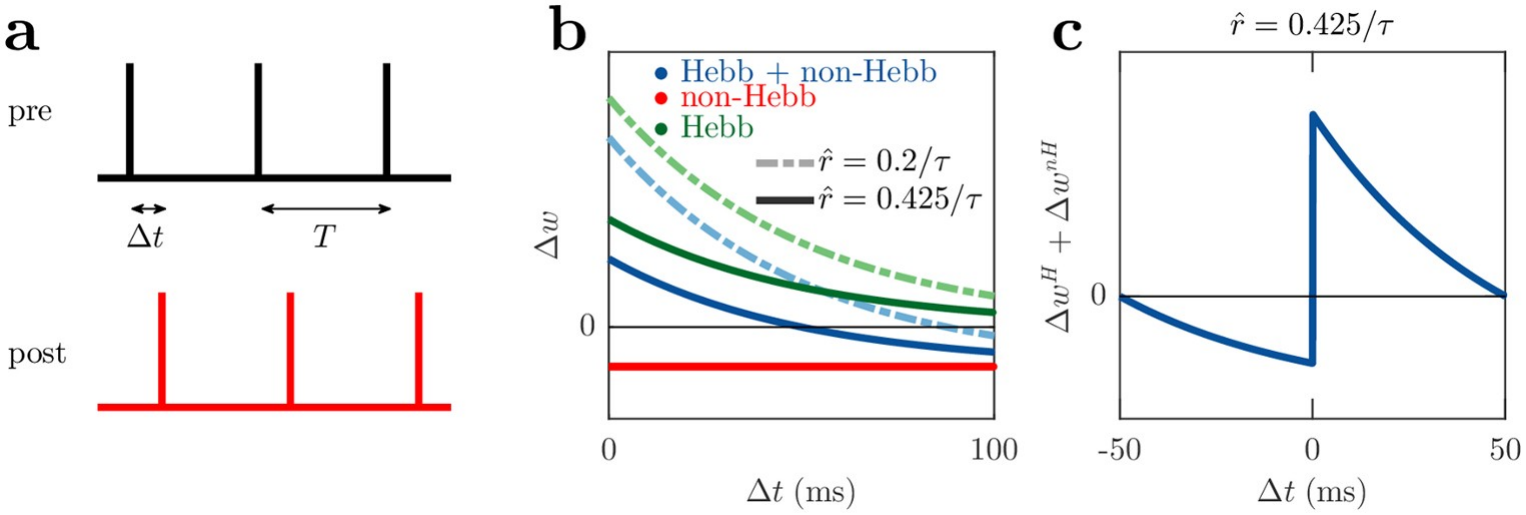
Comparison to STDP. (a) A schematic STDP protocol where presynaptic and postsynaptic spikes are repeated with period *T* and delay Δ*t*. (b) Weight change dependence on the delay between pre and post spikes, for the Hebbian Δ*w*^*H*^ (green), non-Hebbian Δ*w*^*nH*^ (red), and combined plasticity rules, Δ*w*^*H*^ + Δ*w*^*nH*^ (blue), assuming constant membrane potential for simplicity. The plotted curves in (b) and (c) are for an experimental protocol of *T* = 5*τ* = 100 ms [7]. (c) The $\hat{r}=0.425/\tau$ Hebbian + non-Hebbian curve in (b), but with delays 50 to 100 ms shifted to -50 to 0 ms for interpreting as post before pre ordering, thus resembling the common presentation of classical STDP.

To reproduce the STDP-like plasticity in our model, we use the same spiking protocol with pairs of spikes repeated at 10 Hz as in [7] (Fig 3a), and consider the changes resulting from the plasticity rules in Eqs (4) and (6). To do this we assume that the membrane potential, *V*(*t*), and the decoded instantaneous presynaptic firing rate, $\hat{r}\left(t\right)$, are constant. The magnitude of the weight change described by our rules depends on the combination of the membrane potential, *V*(*t*), the decoded instantaneous presynaptic firing rate, $\hat{r}$, the connection strength, *w*, the learning rate, *ϵ*, the capacitance, *C*, and the neuronal time constant, *τ*, in Eqs (4) and (6). The combination of these quantities are not fully constrained by available experimental data so the magnitude of the weight changes in Fig 3b and 3c are arbitrary, but the temporal characteristics are not. The temporal characteristics depend on the relative sizes of the pair stimulation period and the neuronal time constant, *τ*, here we choose a typical value of *τ* = 20 ms [41], one fifth of the 100 ms pair stimulation period.

The non-Hebbian weight change, Δ*w*^*nH*^, indicated by the red line in Fig 3b, is constant as it depends only on the time between postsynaptic spikes which is fixed by the pair frequency. The Hebbian weight change, Δ*w*^*H*^, decays exponentially with Δ*t* indicated by the green lines in Fig 3b (see Sec. STDP and triplet comparison and S1 Appendix for details). However, when $\hat{r}\approx 0.425/\tau$, the combined weight change from both plasticity rules is qualitatively similar to the classical form of STDP (Fig 3c), consisting of LTD for postsynaptic before presynaptic spike timing, increasing in magnitude toward Δ*t* = 0, and LTP for presynaptic before postsynaptic spike timing, decreasing as Δ*t* becomes large. When $\hat{r}>0.425/\tau$, LTP transitions to LTD for large positive Δ*t*. This is consistent with reports of LTP changing to LTD for postsynaptic spikes 20 ms after a presynaptic spike [42]. The relative sizes of *τ* and the stimulated pair frequency are also important. When the pair frequency is much larger than *τ*, again LTP transitions to LTD for large positive Δ*t*.

These results are consistent with observations in [6] which report that LTP only occurred in STDP experiments for synapses that were initially weak, but LTD did not obviously depend on initial synaptic strength. In the context of these plasticity rules for the LTP regime (a presynaptic before postsynaptic spike ordering with a small delay), a small initial connection strength indicates $\hat{r}$ is likely to be small. When $\hat{r}$ is small, the positive Hebbian weight change exceeds the negative non-Hebbian weight change, leading to LTP. However, as the initial connection strength increases, then $\hat{r}$ will increase, reducing LTP for strong initial connection strengths, consistent with the experiments. This behaviour can be seen in Fig 3b in which the LTP regime is reduced as $\hat{r}$ increases.

For postsynaptic before presynaptic spike ordering and the LTD regime, the Hebbian plasticity change is small due to its exponential decay with the delay between presynaptic and postsynaptic spiking. In this case the non-Hebbian plasticity change dominates, meaning that LTD in this regime does not depend on initial connection strength as observed in the experiments. This behaviour can also be seen in Fig 3b for large Δ*t* where LTD dominates and is less affected by changes to $\hat{r}$ compared to the LTP regime.

### Frequency dependence of plasticity

**Fig 4:**
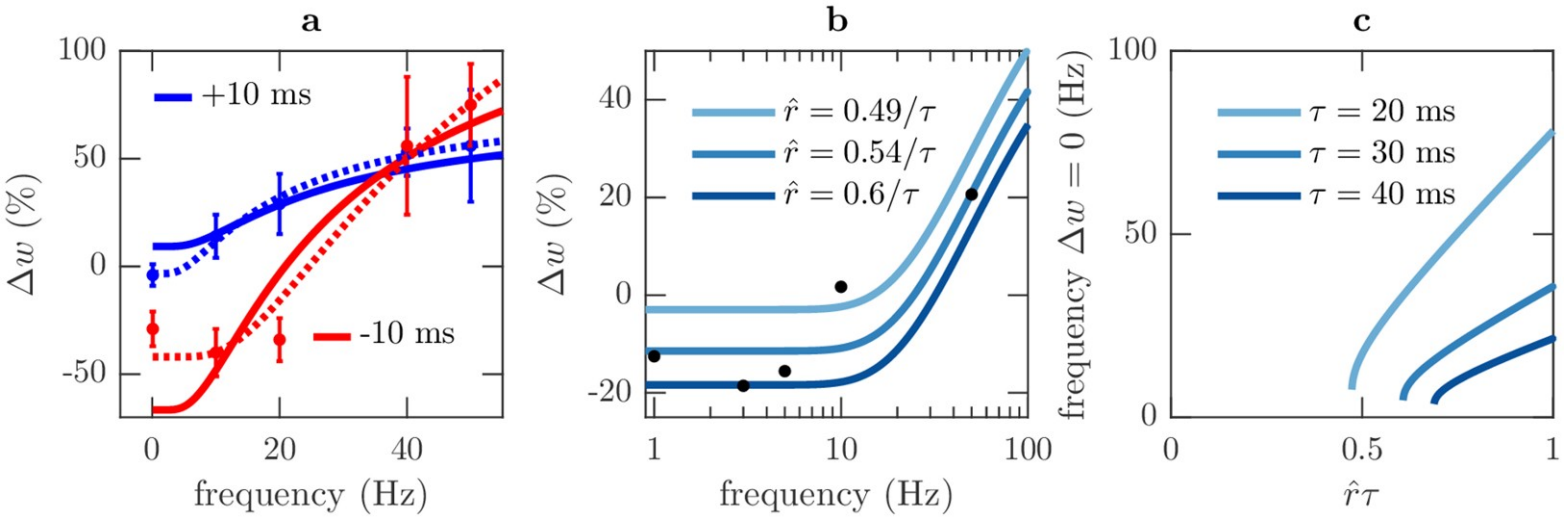
Frequency dependence of plasticity. Details of the curves and parameters are in Sec. STDP and triplet comparison, S1 Appendix and Table 1 (a) In an STDP experimental protocol the combined Hebbian, Δ*w*^*H*^, and non-Hebbian, Δ*w*^*nH*^, plasticity rules (curves) predict a weight change dependence on pair frequency in agreement with experimental data (dots with error bars) from rat visual cortex [33]. Blue and red indicate pre before post and post before pre timing respectively, with a 10 ms delay. Solid lines have identical parameters (aside from $\hat{r}$), while parameters are allowed to vary between spike orderings for dashed lines. (b) Comparison of model to experiments measuring the change in induced response after stimulating rat CA1 at different frequencies [34]. The decoded instantaneous presynaptic firing rate, $\hat{r}$, controls the frequency at which LTD transitions to LTP, similar to the sliding threshold in BCM. (c) Curves showing the model’s behaviour is similar to the BCM sliding threshold mechanism [43] where the transition frequency from LTD to LTP varies with the decoded instantaneous presynaptic firing rate, $\hat{r}$. When $\hat{r}$ is small only LTP occurs so there is no transition between LTD and LTP. As $\hat{r}$ increases, LTD begins at low frequencies and increases further with $\hat{r}$. A time delay of 15 ms between presynaptic and postsynaptic activity is assumed.

STDP experiments in rat visual cortex document a dependence of plasticity on the frequency of spike pairs where LTP is promoted at high frequencies [33, 40]. Our plasticity model reproduces this same frequency dependent promotion of LTP, as shown in Fig 4a (see Sec. STDP and triplet comparison, and S1 Appendix for details of the model curves and Table 1 for parameter values).

**Table 1:** Parameter values used in the comparisons to experimental data.

Fig.	Eq.	Description	Parameter Values
Fig 4a		STDP frequency dependence:	Δ*t* = 10 ms
			$\epsilon n\frac{\left(V_{i}-V_{E/I}\right)}{C}=16.4\;{\text{ms}}^{-1}$
			*τ* = 40 ms
	(9)	pre before post (solid line)	$\hat{r}=0.69/\tau$
	(10)	post before pre (solid line)	$\hat{r}=0.56/\tau$
Fig 4a	(9)	STDP frequency dependence:	Δ*t* = 10 ms
		pre before post (dashed line)	$\epsilon n\frac{\left(V_{i}-V_{E/I}\right)}{C}=14.6\;{\text{ms}}^{-1}$
			*τ* = 57.8 ms
			$\hat{r}=0.88/\tau$
Fig 4a	(10)	STDP frequency dependence:	Δ*t* = 10 ms
		post before pre (dashed line)	$\epsilon n\frac{\left(V_{i}-V_{E/I}\right)}{C}=21.0\;{\text{ms}}^{-1}$
			*τ* = 20 ms
			$\hat{r}=0.25/\tau$
Fig 4b	(9)	Induced response stimulation frequency dependence	Δ*t* = 15 ms
			$\epsilon n\frac{\left(V_{i}-V_{E/I}\right)}{C}=4389\;{\text{ms}}^{-1}$
			*τ* = 20 ms
			$\hat{r}=\left\{0.49,0.54,0.6\right\}/\tau$
Fig 5a			$\epsilon n\frac{\left(V_{i}-V_{E/I}\right)}{C}=0.41\;{\text{ms}}^{-1}$
			*τ* = 50 ms
	(11)	post, pre, post triplets	$\hat{r}=0.05/\tau$
	(12)	pre, post, pre triplets	$\hat{r}=0.37/\tau$

This frequency dependence is a prediction of our model strongly attributed to the spike timing dependence of the non-Hebbian plasticity rule, see Eq (6). For presynaptic before postsynaptic spike pair ordering with Δ*t* = 10 ms as in [33] (Fig 4a blue curve), the frequency dependence of our plasticity rules arises from the reduction in time between successive postsynaptic spikes as frequency increases. This shorter time period reduces the interval of the integral in Eq (6) and thus reduces the depressive non-Hebbian plasticity change, Δ*w*^*nH*^. This frequency dependence also applies to postsynaptic before presynaptic spike pair ordering with Δ*t* = −10 ms as in [33] (Fig 4a red curve); however, in addition, the time between presynaptic and postsynaptic spikes is now dependent on the time between adjacent pairs, not only Δ*t*. At low frequencies there is a large time delay between pairs and therefore the Hebbian plasticity change, Δ*w*^*H*^, is small and the depressive non-Hebbian plasticity change, Δ*w*^*nH*^, dominates. However, as the frequency increases, the time delay between pairs shortens and the Hebbian plasticity change, Δ*w*^*H*^, becomes larger and dominates.

Related experiments stimulated rat CA1 presynaptic neurons at different frequencies, inducing a postsynaptic response and plasticity [34]. The resulting plasticity changes also show that LTP is promoted at high stimulation frequencies. These experiments do not report individual spike timings to which we can directly apply our plasticity rules; however, the frequency dependence can be captured by approximating the spiking activity of a pair of presynaptic and postsynaptic neurons as a series of stimulated presynaptic spikes each followed after a Δ*t* delay by an induced postsynaptic spike, akin to an STDP protocol. Then, using the same assumptions for the STDP experiments described above, the model captures the frequency dependence observed in experiment (see Sec. STDP and triplet comparison, and S1 Appendix for details of the model curves and Table 1 for parameter values).

Additional experimental results have found this frequency dependence is shifted to lower frequencies when rats or mice are raised in darkness, compared to a normal light/dark cycle [1, 35]. This has been interpreted as evidence for the sliding threshold mechanism in the Bienenstock, Cooper and Munro (BCM) model of plasticity, by associating dark rearing with reduced postsynaptic activity [43]. In our model the frequency at which the transition between LTD and LTP occurs changes with the decoded instantaneous presynaptic firing rate, $\hat{r}$, (Fig 4b and 4c) because $\hat{r}$ determines the ratio of the positive Hebbian changes to the negative non-Hebbian changes as described in Eq (2). When the decoded instantaneous presynaptic firing rate, $\hat{r}$, is low the transition frequency is low, promoting LTP and inducing increased postsynaptic activity. As the decoded instantaneous presynaptic firing rate, $\hat{r}$, increases, the transition frequency increases promoting LTD and constraining postsynaptic activity. In the context of light and dark rearing, we predict that the decoded instantaneous presynaptic firing rate, $\hat{r}$, values will be higher for a normal light/dark cycle, indicating a better encoding of information than for dark rearing. Thus, we expect the transition between LTD and LTP to occur at lower frequencies in animals raised in darkness, as is found experimentally.

### Spike triplet protocols

**Fig 5:**
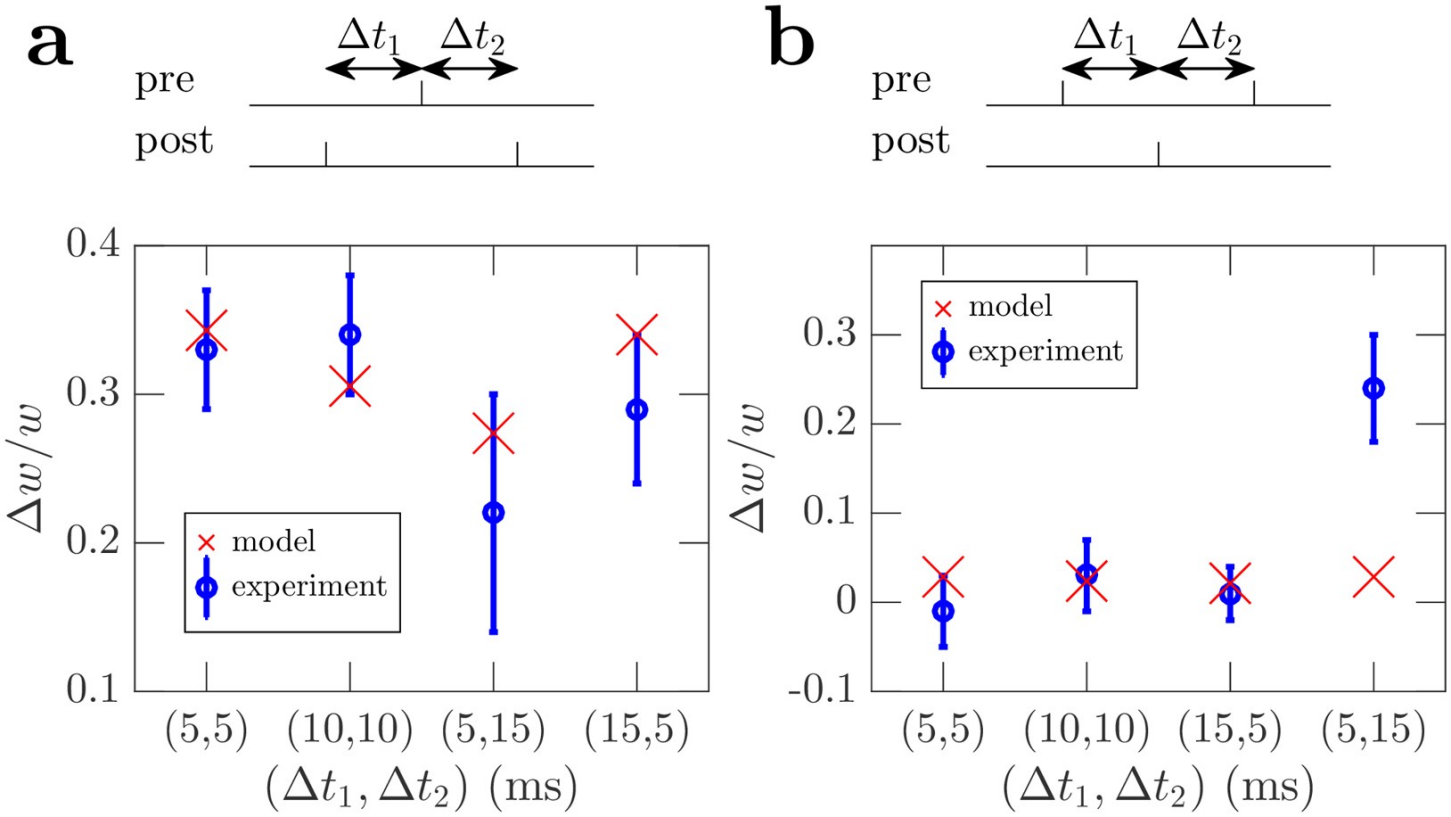
Comparison of experimental weight changes with model predictions for neurons stimulated using triplet protocols with a range of delays between spikes, (Δ*t*_1_, Δ*t*_2_). Blue error bars are experimental data from [44], red crosses are model predictions. For details of the model predictions see Sec. STDP and triplet comparison, S1 Appendix and Table 1. (a) Postsynaptic, presynaptic, postsynaptic spike ordering. (b) Presynaptic, postsynaptic, presynaptic spike ordering.

Work on STDP has been extended experimentally and in models beyond pairs of spikes to consider triplets of spikes consisting of either two presynaptic spikes and one postsynaptic spike, or one presynaptic spike and two postsynaptic spikes [25–27, 44]. We use the same triplet protocols as in [44] to compare model predictions with experimental data of synaptic changes resulting from spike triplet stimulation experiments. These consist of two presynaptic spikes and one postsynaptic spike, or one presynaptic spike and two postsynaptic spikes, repeated at a frequency of 1 Hz and with delays between spikes varying for different trials. Using the same assumptions as for the STDP comparison in Sec. Comparison to classical STDP (see Sec. STDP and triplet comparison, S1 Appendix for details of the model curves and Table 1 for parameter values), our model predicts plasticity changes that are consistent with the experimental results, aside from the (5, 15) ms presynaptic, postsynaptic, presynaptic triplet, see Fig 5. This is similar to the triplet model in [25] which also fit well to these same experimental data, aside from this same data point.

Our plasticity rules are not triplet based; however, a single application of both the Hebbian and non-Hebbian plasticity rules involves three spikes. Two postsynaptic spikes are involved in the non-Hebbian rule. One of those postsynaptic spikes, plus an additional presynaptic are involved in the Hebbian rule.

### Self-organization of a spatially extended spiking neural network with complex real-world stimulus

**Fig 6:**
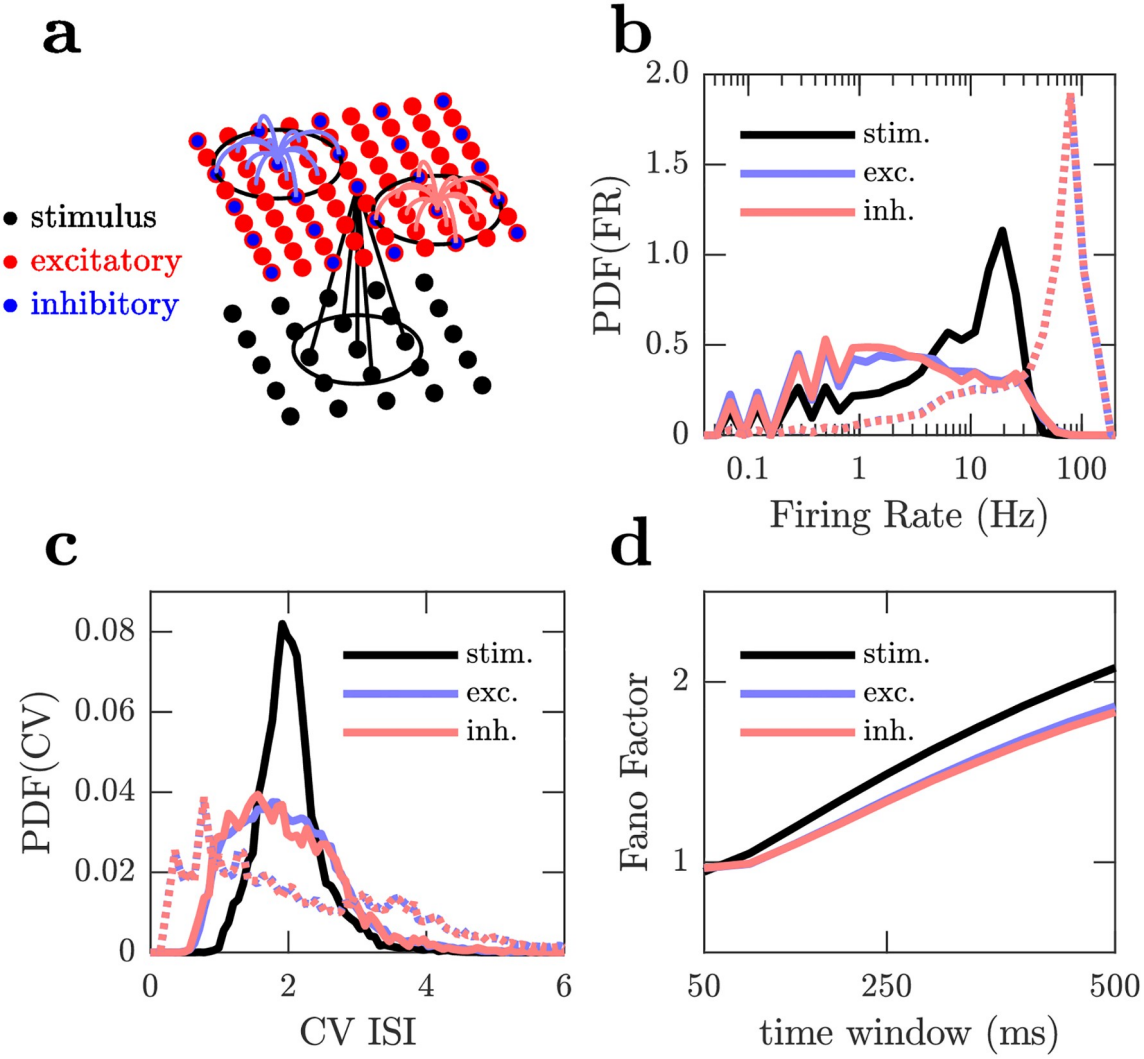
Spiking statistics of the stimulus and neural populations. (a) The demonstration network consists of populations of excitatory (red dots), inhibitory (blue dots) and stimulus neurons (black dots). Each neuron connects to neighbours within a fixed range indicated by black circles. Example input connections to single neurons are shown from excitatory (red lines), inhibitory (blue lines) and stimulus (black lines) populations. All synapses are plastic. (b), (c), (d) Spike statistics recorded during a single pass through the dataset for the stimulus (black), excitatory (blue) and inhibitory populations (red), at the beginning of the simulation (dashed lines) and after a 3100 seconds of learning (solid lines). At the beginning of the simulation the network is initialised with very strong synapses from the stimulus which leads to very high firing rates and low variability. After learning variability increases and firing rates decrease. All distributions are across neurons in each population. (b) Distributions of population coefficients of variation of interspike intervals. (c) Distributions of population firing rates. (d) Population Fano-factors after 3100 seconds of learning.

We now demonstrate that these plasticity rules can be used in recurrent neural populations to encode a high dimensional, rapidly time varying, complex real-world stimulus, approximating the sensory encoding task that real brains must perform. In the process of learning this task, the interplay of our plasticity rules with neural activity gives rise to realistic properties of the circuit dynamics and connectivity. We apply the Hebbian and non-Hebbian plasticity rules in Eqs (4) and (6) to a biologically plausible neural circuit composed of leaky integrate and fire (LIF) neurons with conductance based synapses arranged in a 2D spatial grid consisting of an excitatory and an inhibitory population [17]. These neural populations receive input from a stimulus population whose spiking activity is given by an event based, complex, real-world visual stimulus collected using a Dynamic Vision Sensor (DVS) (see Fig 6a, Table 2 and Methods) [45].

**Table 2:** Numerical values of the parameters used in the simulation. D, E and I denote the stimulus, excitatory and inhibitory populations respectively.

Model Parameter	Symbol	Value
$\hat{r}$ scaling factor: stimulus to excitatory	*γ*^*DE*^	6.83 × 10^−4^ mV^−1^ ms^−1^
$\hat{r}$ scaling factor: stimulus to inhibitory	*γ*^*DI*^	2.73 × 10^−3^ mV^−1^ ms^−1^
$\hat{r}$ scaling factor: excitatory to excitatory	*γ*^*EE*^	1.67 × 10^−3^ mV^−1^ ms^−1^
$\hat{r}$ scaling factor: excitatory to inhibitory	*γ*^*EI*^	6.67 × 10^−3^ mV^−1^ ms^−1^
$\hat{r}$ scaling factor: inhibitory to excitatory	*γ*^*IE*^	2.78 × 10^−4^^−3^ mV^−1^ ms^−1^
$\hat{r}$ scaling factor: inhibitory to inhibitory	*γ*^*II*^	1.11 × 10^−3^ mV^−1^ ms^−1^
learning rate: stimulus to excitatory	*ϵ*^*DE*^	1.25 × 10^−6^ *μ*S mV^−1^
learning rate: stimulus to inhibitory	*ϵ*^*DI*^	1.25 × 10^−6^ *μ*S mV^−1^
learning rate: excitatory to excitatory	*ϵ*^*EE*^	5.12 × 10^−7^ *μ*S mV^−1^
learning rate: excitatory to inhibitory	*ϵ*^*EI*^	5.12 × 10^−7^ *μ*S mV^−1^
learning rate: inhibitory to excitatory	*ϵ*^*IE*^	1.13 × 10^−6^ *μ*S mV^−1^
learning rate: inhibitory to inhibitory	*ϵ*^*II*^	1.13 × 10^−6^ *μ*S mV^−1^
learning noise: stimulus	$p_{noise}^{D}$	0
learning noise: excitatory	$p_{noise}^{E}$	0.75
learning noise: inhibitory	$p_{noise}^{I}$	0.5
initial connection strength: stimulus to excitatory	$W_{init}^{DE}$	2.20 × 10^−2^ *μ*S ms
initial connection strength: stimulus to inhibitory	$W_{init}^{DI}$	2.20 × 10^−2^ *μ*S ms
initial connection strength: excitatory to excitatory	$W_{init}^{EE}$	1.67 × 10^−5^ *μ*S ms
initial connection strength: excitatory to inhibitory	$W_{init}^{EI}$	4.17 × 10^−6^ *μ*S ms
initial connection strength: inhibitory to excitatory	$W_{init}^{IE}$	2.20 × 10^−4^ *μ*S ms
initial connection strength: inhibitory to inhibitory	$W_{init}^{II}$	5.50 × 10^−5^ *μ*S ms
temporal length of *A*(*t*)	*τ*_*A*_	140 ms
membrane capacitance	*C*	1 nF
leak conductance	*g*_*L*_	50 nS
leak reversal potential	*V*_*L*_	−70 mV
excitatory reversal potential	*V*_*E*_	0 mV
inhibitory reversal potential	*V*_*I*_	−80 mV
spike threshold potential	*V*_*th*_	−55 mV
reset potential	*V*_*rt*_	−70 mV
refractory period	*τ*_*ref*_	5 ms
excitatory synaptic rise time	$\tau_{r}^{E}$	0.5 ms
excitatory synaptic decay time	$\tau_{d}^{E}$	2 ms
inhibitory synaptic rise time	$\tau_{r}^{I}$	0.5 ms
inhibitory synaptic decay time	$\tau_{d}^{I}$	7 ms
simulation timestep	Δ*t*	0.1 ms

Due to the initialisation of strong connections from the stimulus (see Table 2), the initial spiking activity of the neural populations is characterised by very high firing rates, 〈*r*〉 = 53.3 Hz and 〈*r*〉 = 53.6 Hz for the excitatory and inhibitory populations, respectively (Fig 6b). We choose the initial connectivity to highlight the ability of the plasticity rules to stabilise and balance the network, even from a poor initial configuration.

**Fig 7:**
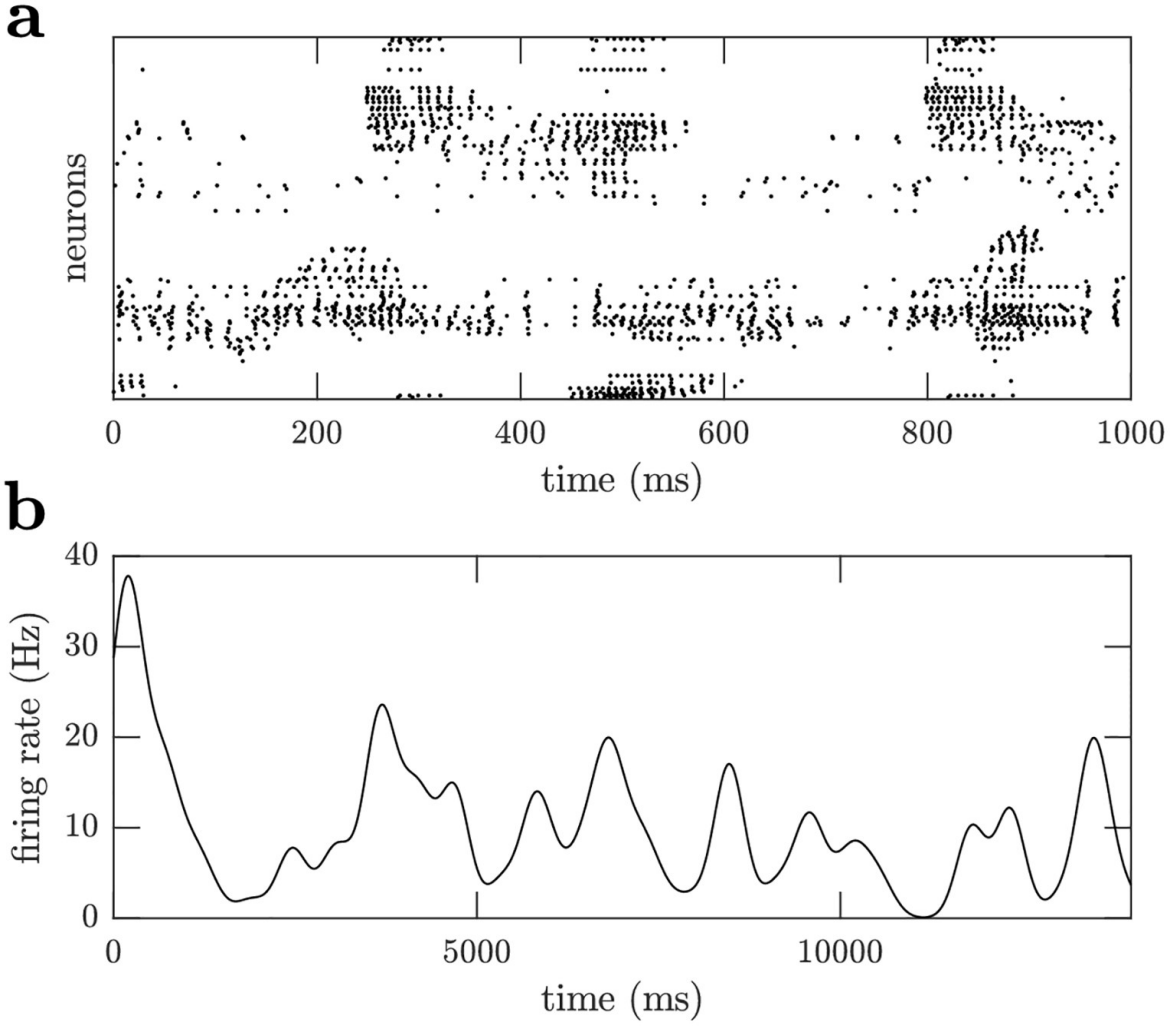
Variability of firing rates. The plasticity rules do not fix the firing rate of neurons, instead firing rates vary in response to the DVS stimulus and recurrent activity. (a) Raster plot of the spiking activity of a sample of excitatory neurons. (b) The firing rate of an excitatory neuron over one pass through the DVS stimulus dataset. The firing rate is obtained by convolving the spike train with a 200 ms standard deviation Gaussian kernel.

Over time the plasticity rules reduce the initially strong connections from the stimulus to the neural populations and increase the strength of the excitatory and inhibitory recurrent feedback. The initial stimulus driven, high firing rate, low variability spiking activity is replaced by activity driven by recurrent excitation and inhibition as well as the stimulus, so that the spike statistics of the neural populations are variable with moderate firing rates; 〈*CV*_*ISI*_〉 = 2.0 (Fig 6c) and 〈*r*〉 = 6.0 Hz (Fig 6b) for the excitatory population, and 〈*CV*_*ISI*_〉 = 1.9 (Fig 6c) and 〈*r*〉 = 5.9 Hz (Fig 6b) for the inhibitory population, while the Fano-Factor increases monotonically from about one using a 50 ms window to 1.8 using a 500 ms window for all populations (Fig 6d), indicating greater variability than Poisson spiking. Such variable spike timing with fluctuating firing rates have been widely observed in the cortex [17, 18, 46]. The fluctuations of the neural populations are related to that of the stimulus driving them (Fig 7). Locally, the activity of a region of a neural population is partly driven by the activity of the corresponding local region of the stimulus. Thus, the variability of the neural populations tends to reflect that of the stimulus.

**Fig 8:**
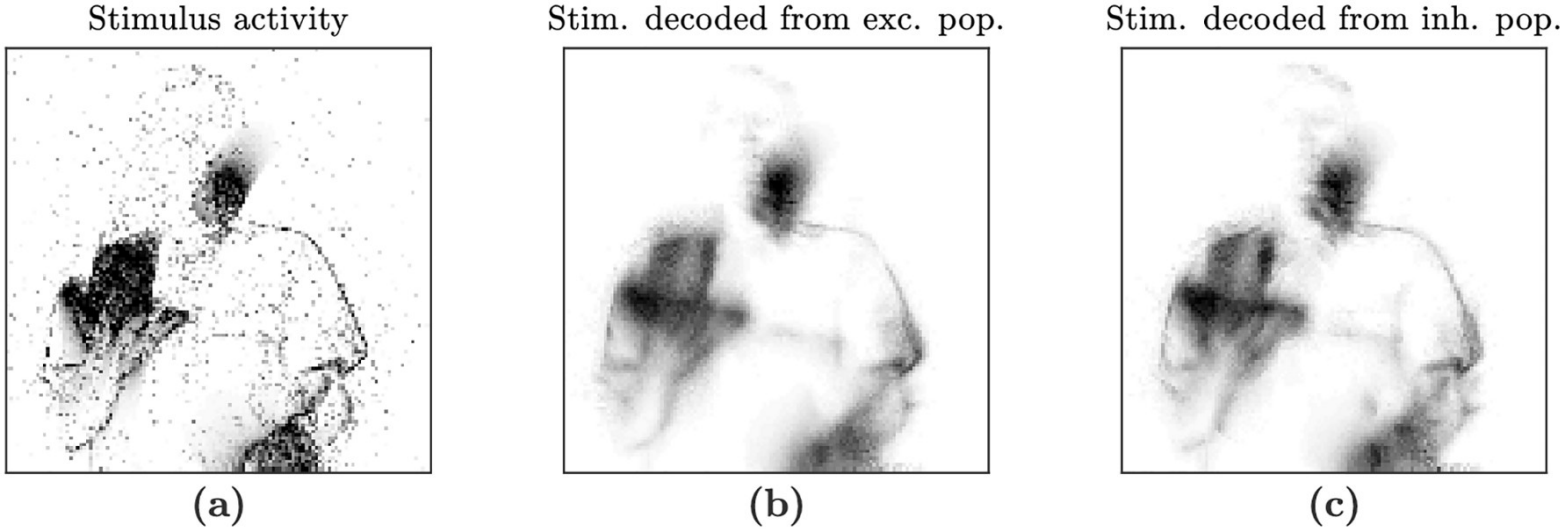
Decoding of the stimulus from each of the neural populations after learning, at one point in time. The stimulus is a recording of a man juggling, viewed front on. An outline of the juggler’s body and the juggling balls are visible in the stimulus itself and in each of the decodings. This shows that the neural populations have learned to encode the stimulus in their activity. This figure is available as a video in S1 Video. (a) DVS stimulus spikes (*r*^*D*^) smeared exponentially in time with membrane potential time constant *τ*, corresponding to the best possible decoding from the neural populations. (b) Decoding of the stimulus instantaneous firing rate from the activity in the excitatory population, ${\hat{r}}^{DE}$. (c) Decoding of the stimulus instantaneous firing rate from the activity in the inhibitory population, ${\hat{r}}^{DI}$.

Comparison of the actual stimulus with the estimates of the stimulus decoded from the activity of the neural populations (Fig 8 and S1 Video) illustrates that the circuit has learned its autoencoder function. In the decoded stimulus, the outline of the moving objects in the time varying input are visible and align with the actual stimulus, whereas in the poor initial configuration before learning, the decoded stimulus is not similar to the actual stimulus.

**Fig 9:**
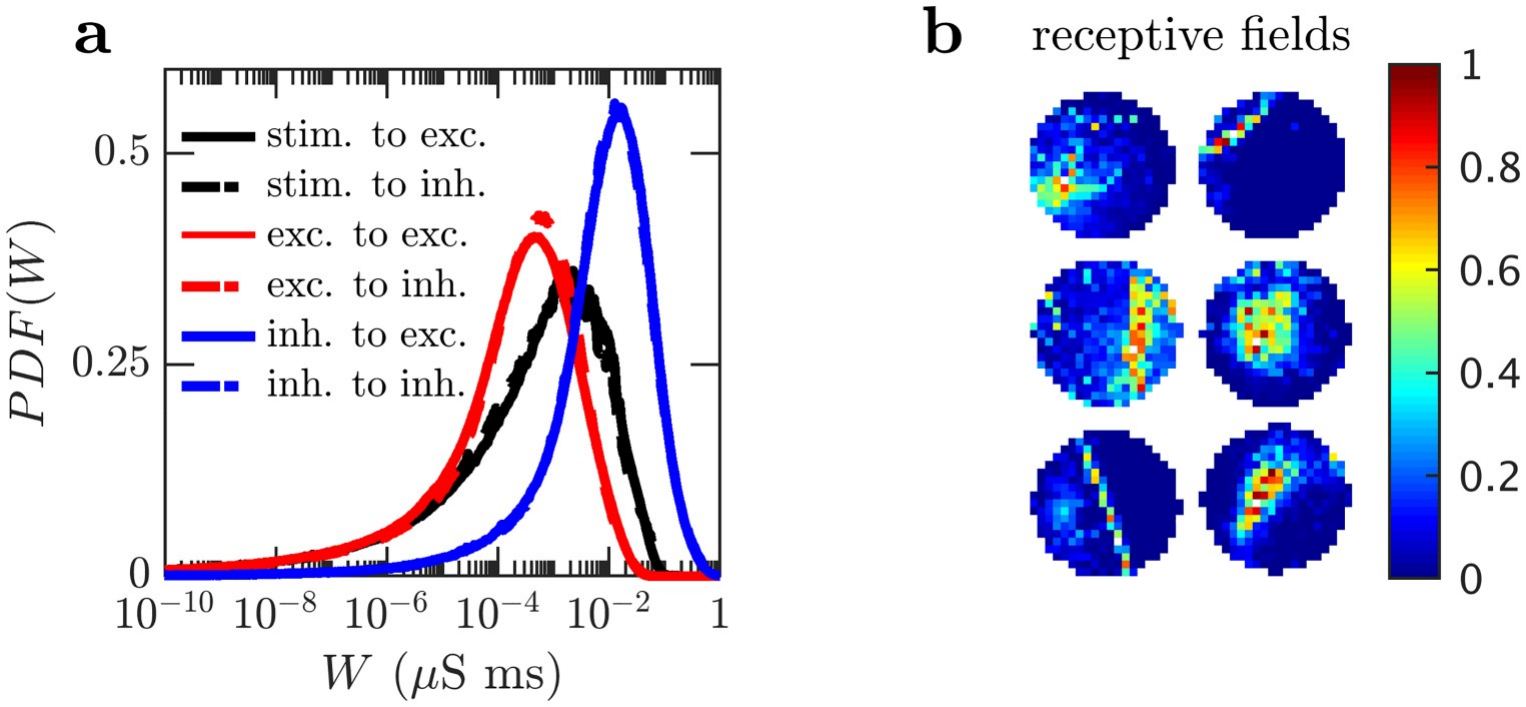
Learned connectivity with DVS stimulus. (a) Long tailed, approximately log-normal, distributions of synaptic strengths. Black curves are excitatory synapses from the stimulus population. Red curves are excitatory synapses from the excitatory population. Blue curves are inhibitory synapses from the inhibitory population. Solid lines indicates synapses with excitatory postsynaptic neurons, dotted lines indicate synapses with inhibitory postsynaptic neurons. (b) Receptive fields (connection strengths from the stimulus to individual neurons) of six excitatory neurons displaying learned selectivity for different features. Red indicates strong synapses, blue indicates weak synapses. Synaptic strengths have been normalized for each neuron.

The resulting synaptic strengths are distributed approximately log-normally (Fig 9a), as has been indicated experimentally [19, 20, 47]. During learning, competition between nearby neurons leads to the development of receptive fields in which the connections from the stimulus to individual neurons adapt so that nearby neurons receive inputs from and respond to different local features of the stimulus (Fig 9b). The development of receptive fields is necessary from a theoretical perspective as these receptive fields indicate that neurons encode features of the input, creating a more useful representation for performing other tasks.

### Emergent stability and balanced excitation and inhibition

Experiments indicate that the excitatory and inhibitory input currents to an individual neuron are close to equal in magnitude or balanced, when averaged over a long time period. Further, tight balance occurs when the currents track each other [16, 21, 48].

We now describe how balanced excitatory and inhibitory currents can emerge using these plasticity rules by first considering stability. When a neural population is stable, on average each new spike that occurs will contribute toward the net production of one further spike distributed amongst its postsynaptic neurons. Thus, if we observe a period of activity containing a combined *s*_*E*_, *s*_*I*_, *s*_*D*_ excitatory, inhibitory and stimulus spikes respectively, then we require that the net contribution from all of these spikes to further spikes be equal to the change in membrane potential required to produce them. Recalling that ${\hat{r}}_{j}^{\alpha \beta }\left(t\right)$ is proportional to the summed changes in membrane potential of its spiking postsynaptic neurons (Eq 3), this condition is described by

$$\begin{array}{c} V_{s}s_{\beta }=s_{E}\frac{\left\langle {\hat{r}}^{E\beta }\right\rangle }{\gamma^{E\beta }}+s_{D}\frac{\left\langle {\hat{r}}^{D\beta }\right\rangle }{\gamma^{D\beta }}-s_{I}\frac{\left\langle {\hat{r}}^{I\beta }\right\rangle }{\gamma^{I\beta }}, \end{array}$$ (8)

where *V*_*s*_ = *V*_*T*_ − *V*_*R*_ is the change in membrane potential from reset to threshold that is required to produce one spike, and $\langle {\hat{r}}^{\alpha \beta }\rangle$ is the average of the decoded instantaneous presynaptic firing rate, ${\hat{r}}^{\alpha \beta }$, over each neuron in the *α* population at their spike times. Each of the terms on the right hand side describe the expected contribution to spikes in *β* from spikes in each of the inhibitory, excitatory and stimulus populations.

The stability condition described by Eq (8) configures learning to produce balanced excitatory and inhibitory currents if the scaling factors, *γ*^*αβ*^, are chosen so that the changes in membrane potential caused by the excitatory and inhibitory currents are large compared to their difference. In S1 Appendix we demonstrate that the learning of this tight balance using our plasticity rules converges because it can be viewed as a gradient descent in a firing rate model.

**Fig 10:**
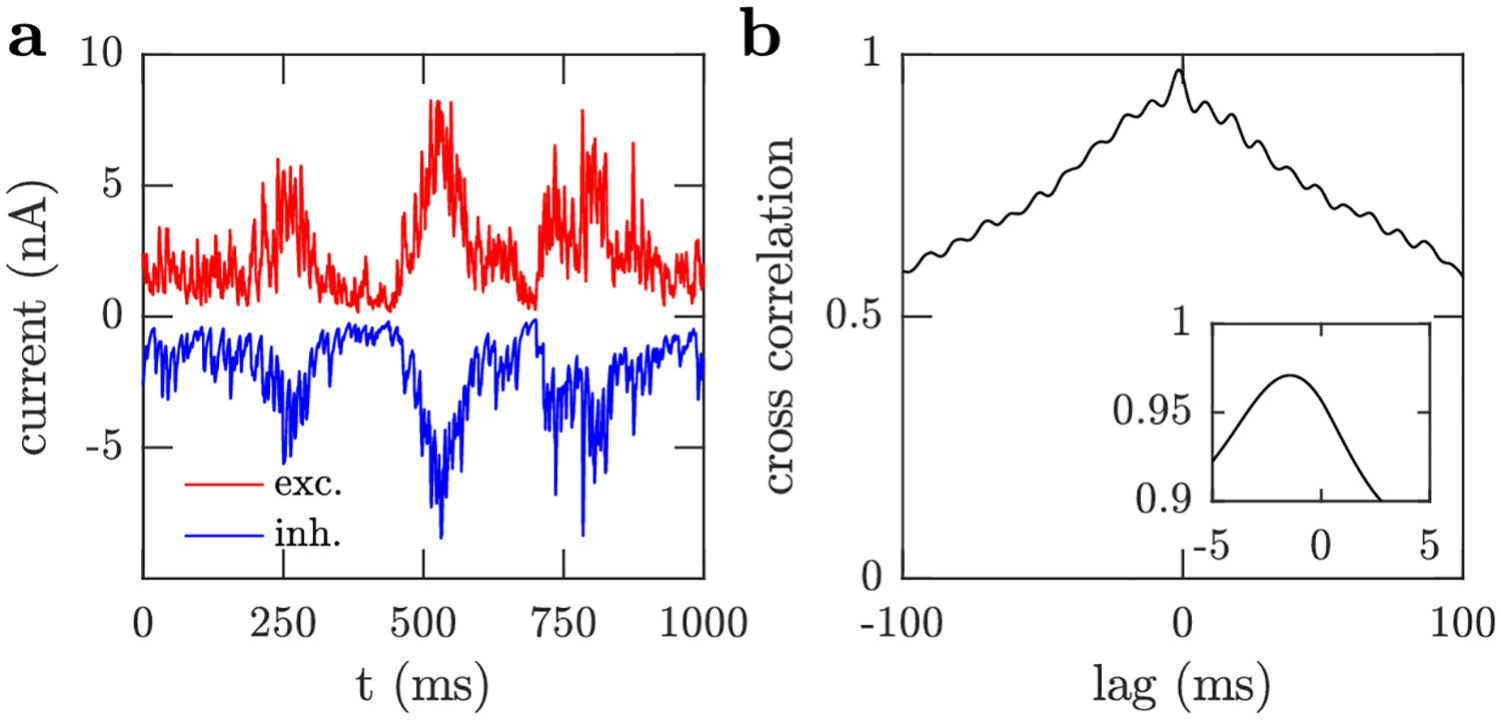
Balance of excitation and inhibition. (a) After learning in the simulation using the DVS stimulus, the excitatory (red) and inhibitory (blue) currents into an example neuron are tightly balanced, with inhibition lagging slightly behind excitation. (b) Cross-correlation between the excitatory and inhibitory currents in (a). The inset corresponds to the central peak with the same axes units. The currents are strongly correlated with a small time lag of 1.5 ms.

In the demonstration circuit stimulated with the DVS stimulus, the plasticity rules are configured by choosing the scaling factors, *γ*^*αβ*^, to satisfy Eq (8) so that the total amount of excitation from the stimulus population is approximately equal to the total amount of excitation from the excitatory population, and this combined excitation is closely tracked and balanced by inhibition (Fig 10a), with a small 1.5 ms delay evident in their cross-correlation (Fig 10b), as found in neural recordings [16].

## Discussion

### Relation to other work

There is a growing body of work attempting to describe and understand the diverse range of experimentally observed features of plasticity, for example [3, 5, 10, 11, 23, 24, 49]. Some past studies combine experimentally motivated plasticity rules and apply them to neural circuits. The parameters of these models are then tuned in order to produce behaviour that is then argued to support some kind of function such as memory or decision making [23, 26–30]. However, this approach does not adequately connect plasticity to the broader, more important picture of why plasticity exists at all; perhaps most importantly, for learning function. In theoretical models, learning implemented via plasticity requires carefully constructed plasticity rules whose properties are governed by an overarching learning algorithm. Thus, an adequate theoretical understanding of plasticity requires a description of its functional role in an overarching learning algorithm. In contrast to previous observation driven models, we have proposed an approach that explicitly links the learning of function to the underlying plasticity phenomena. We *started* by considering how to learn function and then *find* that this leads to plasticity rules that have properties similar to that observed experimentally, thus directly explaining and connecting these plasticity phenomena to their functional roles in learning.

More specifically, we have investigated a description of plasticity derived from extending the classical autoencoding function used in machine learning to recurrent networks in which individual neurons learn to encode their own activities into the population activity. This necessitates both Hebbian and non-Hebbian plasticity rules as they each account for the spiking statistics required for autoencoding, contrasting with a variety of other plasticity models [5, 24] where the use of Hebbian and non-Hebbian rules is motivated primarily by stability considerations [23] and to align with experimental observations, for example [25, 50].

These Hebbian and non-Hebbian rules are related by the presynaptic neuron’s instantaneous firing rate decoded from the population activity. This relationship entails a dependence on past activity. In many past studies, for example [12–14], the dependence of plasticity rules on past activity has been termed metaplasticity. Thus, in this context the relationship can be considered a form of metaplasticity. However, it should be noted that the relationship is integral to the plasticity rules themselves and is not separate from them, as the term metaplasticity may imply. This extends the existing understanding of metaplasticity by theoretically describing its interaction with plasticity, learning and function, whereas previously metaplasticity has mostly been investigated from an experimental perspective and considered as a mechanism to regulate synaptic strengths and prevent them from saturating [12–14].

The decoded instantaneous firing rate varies at short timescales with changes in postsynaptic activity, but also varies at longer timescales with the gradual, accumulated changes in connection strengths due to plasticity. These variations at multiple timescales explain how fast but unstable Hebbian plasticity can be stabilised by homeostatic plasticity that experimentally appears slow, but also works at faster timescales. This resolves the paradox of the theoretical need for fast homeostatic plasticity to stabilise unstable Hebbian plasticity with experimental observations of considerably slower homeostatic plasticity evolving over hours or days [5, 10, 11].

The form of our plasticity rules capture the exponential spike timing dependence of classical STDP as well as the frequency dependence of both STDP and non-timing based stimulation experiments, including a sliding threshold like mechanism in which the decoded instantaneous firing rate controls the frequency at which LTD transitions to LTP. Spike-triplet models have also been investigated as a means of providing a spike timing based account of BCM [51], and have been investigated for possible functional roles, such as maximising information transmission [52, 53], or bounds on differences between target and model distributions [54].

In our model of plasticity, the stability of recurrent neural circuits is linked to their function because the learned quantity for each neuron, its decoded instantaneous firing rate, is proportional to the gain of that neuron. Furthermore, gains between populations can be controlled in this same way by decoding from each population separately. When our plasticity rules are implemented in a neural circuit, balancing these gains between excitatory and inhibitory populations at a network level leads to tightly balanced excitatory and inhibitory currents into individual neurons, which has been observed in experiments [21]. Other models of plasticity have produced this balance [23, 55], but have not made the connection to a range of other plasticity phenomena, as described above.

This tight balance is thought to explain variability in spiking activity of individual neurons because spikes are generated by an imbalance over short time windows. Consistent with experimental observations of spiking variability, we also find that these plasticity rules are able to shift a network from a deliberately poor initial regime of very strong stimulus input driving high firing rates, to a balanced regime driven by recurrent activity in conjunction with a much weakened stimulus input. In this regime, firing rates are reduced and neurons exhibit variable spiking. The plasticity rules do not enforce a fixed firing rate, unlike other models [23], rather firing rates fluctuate in response to the stimulus and recurrent dynamics so that the neurons can perform their autoencoding function.

The self-organising process in the interplay between the neural circuit and its plasticity mechanisms also accounts for other salient features of neural dynamics including approximately log-normal distributions of connection strengths, as has been indicated experimentally [19, 20]. Neurons develop receptive fields that extract features optimised for the particular stimulus used in this study. These emergent receptive fields enable the complex, real-world visual stimulus to be accurately decoded from the neural populations, indicating that the original function underpinning these plasticity rules was learned.

Our plasticity rules are directly applicable to implementing autoencoder learning in neural circuits stimulated with complex, real-world data. The function of the neural circuit can be directly observed by decoding the circuit’s activity. This is unlike many other models that apply plasticity rules that are not derived from function, or are derived from functional principles but are not linked to a task and therefore have an unclear relationship between their resulting neural dynamics and implementing brain functions [23, 26–30].

### Experimental predictions and limitations

These results provide theoretical motivation for revisiting previous plasticity experiments to more closely examine past interpretations, as expressed elsewhere [24, 40, 56]. In particular, these results imply that classical STDP is a special case of a combination of more fundamental plasticity rules, and that non-Hebbian plasticity should also exhibit a spike timing dependence on postsynaptic spikes in individual neurons. This non-Hebbian spike timing dependence can explain the frequency dependence of plasticity, though we are not presently aware of any existing attempts at directly observing non-Hebbian spike timing dependence.

To date, many plasticity experiments tend to focus on controlling a single variable, often spike timing, and measuring synaptic changes. However, it is very likely that many neuronal and synaptic variables, not just spike timing, are strongly implicated in brain function and therefore in learning and plasticity. Thus, plasticity rules are likely to depend on a range of variables that are not usually simultaneously reported for individual neurons in experimental work. In the case of our model, since our Hebbian plasticity rule strongly depends on the decoding of the presynaptic activity, to compute this it is necessary to measure the connection strengths, membrane potentials and spiking activity of all postsynaptic neurons to gain a more comprehensive understanding of synaptic plasticity. These properties are likely to vary greatly between individual neurons, thus without such measurements, comparing our model to data requires making assumptions about these unmeasured variables and introducing unconstrained parameters. Further, the potential variability between neurons may mean that averaging across a small number of neurons could be misleading and a potential source of conflicting observations [1, 6, 7, 44, 57]. More complete experimental characterisations of plasticity will require fewer assumptions to be made when comparing to models.

Further to this, fewer experiments have studied plasticity of inhibitory synapses than excitatory ones; however, these experiments show an inconsistent range of different timing based behaviours of inhibitory synapses that differ to those observed for excitatory synapses [58]. Thus, further experiments are required to determine if these differences between excitatory and inhibitory plasticity observations are a result of different experimental designs or differences in the underlying plasticity rules. The plasticity rules we present apply to both excitatory and inhibitory synapses, meaning that broadly they share the same timing and frequency features, but the synaptic reversal potential changes depending on the synapse type, thus excitatory and inhibitory plasticity have a different dependence on postsynaptic membrane potential.

Plasticity rules must be local, meaning that modifying a synapse does not require detailed information about distant parts of the network. It is often assumed that plasticity rules can only use information about neurons on either end of a synapse [10]; however, from a functional perspective this assumption is very restrictive as it limits the ability of connections to organise collectively. In contrast, our Hebbian rule involves the decoded instantaneous firing rate which is computed using information about all postsynaptic spiking neurons. Thus, our plasticity rules while still local, have locality relaxed to include information not only about both the presynaptic and postsynaptic neurons on either end of a synapse, but also other neurons that are postsynaptic to the presynaptic neuron.

We do not yet have a mechanistic description of how these plasticity rules are implemented in biology. Discovering a mechanism by which postsynaptic signals can be integrated and distributed to synapses would be an important step in demonstrating the biological plausibility of this relaxed locality. Further to this, a detailed description of the underlying biological mechanisms would enable a more informed comparison to experiment. For example, some plasticity experiments isolate target neurons by removing surrounding neurons to which the target neurons are likely connected [44]. At present it is not clear if removing these neurons is equivalent to their connections never being present at all, or if underlying mechanisms are disrupted in this process, leading to spurious results. Further, it is not clear if external control over the activity of neurons disrupts these underlying mechanisms, for example as in STDP experiments.

### Conclusion

In conclusion, based on the function of neurons learning to encode their activity into the population activity so that neural circuits learn to act as recurrent autoencoders, we present a novel, unified account of synaptic plasticity, dynamics and neural coding that explains a great variety of plasticity features and neural dynamics. This unified account thus represents a significant advance toward understanding the working mechanisms of cortical circuits. Further explanations of how such emergent plasticity rules and their interplay with neural circuits can explain cognitive functions would be an important direction for future studies.

## Methods

### STDP and triplet comparison

To compare our model to STDP and triplet experiments we apply the plasticity rules in Eqs (4) and (6) according to the timings of the spikes, as described in S1 Appendix. The resulting equations describing the expected weight change are as follows.

For STDP presynaptic before postsynaptic timing

$$\begin{array}{c} \frac{\Delta w_{ij}}{w_{ij}}=\mp \epsilon n\frac{V_{i}-V_{E/I}}{C}(\frac{e^{-\Delta t/\tau }}{{\hat{r}}_{j}}-\tau (1-e^{-T/\tau })). \end{array}$$ (9)

For STDP postsynaptic before presynaptic timing

$$\begin{array}{c} \frac{\Delta w_{ij}}{w_{ij}}=\mp \epsilon n\frac{V_{i}-V_{E/I}}{C}(\frac{e^{-(T-\Delta t)/\tau }}{{\hat{r}}_{j}}-\tau (1-e^{-T/\tau })). \end{array}$$ (10)

For postsynaptic, presynaptic, postsynaptic triplets

$$\begin{array}{c} \frac{\Delta w_{ij}}{w_{ij}}=\mp \epsilon n\frac{V_{i}-V_{E/I}}{C}(\frac{e^{-\Delta t_{2}/\tau }}{{\hat{r}}_{j}}-\tau (2-e^{-(\Delta t_{1}+\Delta t_{2})/\tau })). \end{array}$$ (11)

For presynaptic, postsynaptic, presynaptic triplets

$$\begin{array}{c} \frac{\Delta w_{ij}}{w_{ij}}=\mp \epsilon n\frac{\left(V_{i}-V_{E/I}\right)}{C}\left(\frac{e^{-\Delta t_{1}/\tau }}{{\hat{r}}_{j}}-\tau \right). \end{array}$$ (12)

### A spatially extended, conductance-based spiking neural circuit

The spiking neural circuit model is based on that used in [17] and is summarised in Tables 2 and 3. We consider a 2D network of 300 × 300 coupled, conductance-based leaky integrate-and-fire neurons consisting of 80% excitatory and 20% inhibitory neurons, as well as a population of stimulus neurons whose spiking activity is controlled by an external data source (Fig 6a). Both excitatory and inhibitory neurons are evenly spaced, with the spacing between inhibitory neurons twice the spacing between excitatory neurons. Connections exist between all pairs of neurons within 15 grid units of each other.

**Table 3:** Simulation model summary.

Populations	Excitatory stimulus (D), excitatory (E), and inhibitory (I)
Topology	2D square Cartesian grid without periodic boundaries. Each population is overlaid on the same 300 × 300 grid unit 2D plane, with the spacing of neurons in each population adjusted so that each population covers this space evenly. Excitatory stimulus population forms a 128 × 128 grid of 16 384 neurons Excitatory population forms a 300 × 300 grid of 90 000 neurons Inhibitory population forms a 150 × 150 grid of 22 500 neurons
Connectivity	All neurons are connected in both directions to neurons in their own and all other populations within a 15 grid unit radius, aside from E to D and I to D connections, which do not exist.
Neuron model	E and I populations: Leaky integrate-and-fire neurons with a fixed threshold and refractory period, see Eq (13). Stimulus (D) population spiking activity is taken from the DVS dataset.
Synapse model	Conductance-based, difference of exponentials synapses, see Eq (15)
Plasticity	Hebbian, see Eq (4). Non-Hebbian, see Eq (6).

We denote the membrane potential of a neuron *i* at time *t* as *V*_*i*_(*t*), with dynamics given by the following:

$$\begin{array}{c} C\frac{d}{dt}V_{i}\left(t\right)=-g_{L}\left[V_{i}\left(t\right)-V_{L}\right]-g_{i}^{E}\left(t\right)\left[V_{i}\left(t\right)-V_{E}\right]-g_{i}^{I}\left(t\right)\left[V_{i}\left(t\right)-V_{I}\right], \end{array}$$ (13)

where the capacitance *C* = 1 nF, the leak conductance *g*_*L*_ = 50 nS, and the reversal potentials are *V*_*L*_ = −70 mV, *V*_*E*_ = 0 mV, and *V*_*I*_ = −80 mV for the leak, excitatory, and inhibitory conductance, respectively [41]. If the membrane potential of a neuron reaches the threshold of *V*_*T*_ = −55 mV, a spike is generated and the membrane potential is reset to the reset potential *V*_*R*_ = −70 mV for a refractory period *τ*_*ref*_ = 5 ms. The synaptic conductances are as follows:

$$\begin{array}{c} g_{i}^{E/I}=\sum_{j}w_{ij}^{E/I}\sum_{k}G^{E/I}\left(t-t_{k}\right), \end{array}$$ (14)

where E and I indicate excitatory and inhibitory respectively, and *t*_*k*_ is the time of the *k*-th spike emitted by the presynaptic neuron *j*. The time course of the postsynaptic conductance is given by the following:

$$\begin{array}{c} G^{E/I}\left(t\right)=\frac{e^{-t/\tau_{d}^{E/I}}-e^{-t/\tau_{r}^{E/I}}}{\tau_{d}^{E/I}-\tau_{r}^{E/I}} \end{array}$$ (15)

with rise times $\tau_{r}^{E}=0.5$ ms and $\tau_{r}^{I}=0.5$ ms, and decay times $\tau_{d}^{E}=2.0$ ms and $\tau_{d}^{I}=7.0$ ms. The denominator is a normalisation factor such that $\int_{0}^{\infty }G^{E/I}\left(t\right)dt=1$. The network model is simulated using a time step of 0.1 ms.

The plasticity rules described by Eqs (4) and (6) are applied to all connections with the parameters listed in Table 2. All connections from the stimulus population to both neural populations are initialised to be very strong, while all connections between the neural populations are initialised to be very weak (Table 2). In this configuration the network activity is driven strongly by the stimulus with little contribution from recurrent connectivity within and between the neural populations.

Code for these simulations is available at https://github.com/BrainDynamicsUSYD/SpikeNet. The file /SpikeNet/cases/generate_learning_config.m is a MatLab script that can be used to generate a configuration file that will instruct the simulator to implement these plasticity rules. Further information on installing and running the simulator can be found in the readme /SpikeNet/README.md.

### DVS dataset

In this neural circuit we include a 128 × 128 population of stimulus neurons whose spiking activity is controlled by an external data source. This population connects feedforward to both the excitatory and inhibitory populations. The stimulus dataset used in this work was recorded using a Dynamic Vision Sensor (DVS) [45]. The DVS is a retina inspired imaging device that captures events rather than frames as in traditional video cameras, and provides a natural spiking stimulus for SNN models, where each pixel corresponds to one neuron. The DVS used to collect the dataset has a resolution of 128x128 pixels, and 1 *μ*s temporal precision, with a minimum 15*μ*s between sequential events for each pixel. This ability to record events at fast timescales makes it much more suitable as a visual input to a SNN model than conventional frame based video cameras whose frame updates are typically every few tens of milliseconds and are much slower than the neuron dynamics that evolve significantly over millisecond timescales.

Each pixel in the DVS records an event when the light intensity on that pixel changes above a threshold value. The DVS is therefore sensitive to movement, changes in lighting or changes in reflective properties of objects. The absence of events means that there has been no change, not that the visual scene is empty. The dataset was obtained from https://www.ini.uzh.ch/∼tobi/dvs/ and is a recording of a person juggling, viewed front on.

### Using noise to reduce over-fitting

Dropout is a simple and effective technique used to reduce over-fitting in artificial neural networks by adding noise to the network through probabilistically deactivating neurons. Dropout can be thought of as a form of model averaging and leads to better generalisation [59].

Here, we also inject noise into the learning scheme to reduce over-fitting; however, unlike in dropout we do not probabilistically omit the transmission of spikes between neurons, but only omit spikes from the learning scheme by omitting applications of plasticity rules involving dropped spikes. Because of this no rescaling of weights is required after learning as in dropout, thus this form of regularisation can be implemented online and is biologically plausible. All spikes are communicated between neurons so this does not represent synaptic failure; however, it would be possible to modify the form of noise or synaptic dynamics to investigate effects like synaptic failure or short term depression and how they may enhance or degrade learning.

## Supporting information

S1 Appendix Further derivations. Additional details underlying the relation between Hebbian and non-Hebbian rules, stability of learning rules, comparisons to STDP and its frequency dependence, spike triplets and the organisation of tightly balanced currents.
(PDF)

S1 VideoVideo version of Fig 8.Decoding of the stimulus from each of the neural populations after learning, at one point in time. The stimulus is a recording of a man juggling viewed front on. An outline of the juggler’s body and the juggling balls are visible in the stimulus itself and each of the decodings. (a) Stimulus spikes smeared exponentially in time with time constant *τ*, corresponding to the best possible decoding from the neural populations, *r*^*D*^. (b) Decoding of the stimulus from the excitatory population, ${\hat{r}}^{DE}$. (c) Decoding of the stimulus from the inhibitory population, ${\hat{r}}^{DI}$.(MPG)
