# Supplementary material for: Functional mechanisms underlie the emergence of a diverse range of plasticity phenomena
Source: PLoS Comput Biol. 2018 Nov 12;14(11):e1006590. doi: 10.1371/journal.pcbi.1006590 (PMC6258383; doi:10.1371/journal.pcbi.1006590)
Supplement: S1 Appendix — Additional details underlying the relation between Hebbian and non-Hebbian rules, stability of learning rules, comparisons to STDP and its frequency dependence, spike triplets and the organisation of tightly balanced currents. (PDF) [file pcbi.1006590.s001.pdf]

Functional mechanisms underlie the emergence of a diverse range of plasticity phenomena.

S1 Appendix. Further Derivations.

James A. Henderson[1,2] and Pulin Gong[1,2]

[1] School of Physics, The University of Sydney, NSW, 2006, Australia.
[2] ARC Centre of Excellence for Integrative Brain Function, The University of Sydney, NSW, 2006, Australia.

Relation Between Hebbian and Non-Hebbian Learning Rules

As described in the main text, to learn $\hat{r}_j(A(t))$ we require both a Hebbian and a non-Hebbian learning rule. We now describe the relation between the Hebbian and non-Hebbian learning rules, starting from Eq. (1) in the main text.

Let ΔR_x be the change to $\hat{r}$ after applying a learning rule (i.e. write $R_x(\hat{r}) = \hat{r} + \Delta R_x(\hat{r})$). Each time $A(t)$ occurs the non-Hebbian rule is applied and produces a change ΔR_{nH}. Recall that r denotes the *instantaneous* firing rate of the presynaptic neuron (given $A(t)$). Thus, each time $A(t)$ occurs the expected number of presynaptic spikes and corresponding applications of R_H is Δtr, giving an expected Hebbian change of $\Delta tr \Delta R_H$. Using this we can rewrite Eq. (1) to give an expected value after N occurrences of $A(t)$

$$\hat{r}^N = \left[\Delta R_{nH}(\hat{r}^{N-1}) + \Delta tr \Delta R_H(\hat{r}^{N-1})\right] + ... + \left[\Delta R_{nH}(\hat{r}^0)) + \Delta tr \Delta R_H(\hat{r}^0)\right] + \hat{r}^0, \tag{S.1}$$

where as in Eq. (1) the brackets group the Hebbian and non-Hebbian learning rules for each occurrence of $A(t)$, and for ease of reading we have dropped the j subscripts and the $A(t)$ argument for $\hat{r}$.

For biological plausibility we require that learning be done online simultaneously with the network performing its function, that is we do not allow different periods of activity for learning and function. This means that the application of the learning rules can not cause $\hat{r}^N$ to significantly deviate from the desired value, so we require $\hat{r}^N \approx \hat{r}^0 = r$, for any N. Using this requirement we can write

$$\hat{r} = [\Delta R_{nH}(\hat{r}) + \Delta t\hat{r} \Delta R_H(\hat{r})] + ... + [\Delta R_{nH}(\hat{r})) + \Delta t\hat{r} \Delta R_H(\hat{r})] + \hat{r}. \tag{S.2}$$

To satisfy this equation we require

$$\Delta R_{nH} = -\Delta t \hat{r} \Delta R_H. \tag{S.3}$$

This corresponds to Eq. (2) in the main text.

Stability of Learning Rules

Below we explain why the stability of the learning scheme requires the Hebbian learning rule to slightly increase synaptic strengths, while the non-Hebbian learning rule slightly decreases synaptic strengths.

The relation between the Hebbian and non-Hebbian learning rules is described by Eq. (2), but this does not specify which of the Hebbian and non-Hebbian rules should be positive or negative. To determine this we consider the stability of learning. Consider now the situation where $\hat{r} \neq r$. The expected change to $\hat{r}(A(t))$ after an occurrence of $A(t)$ is

$$\langle \Delta \hat{r} \rangle = \Delta R_{nH}(\hat{r}) + \Delta t r \Delta R_H(\hat{r}). \tag{S.4}$$

Using Eq. (2) we have

$$\langle \Delta \hat{r} \rangle = (r - \hat{r}) \Delta t \Delta R_H(\hat{r}). \tag{S.5}$$

If $r > \hat{r}$ we want $\langle \Delta \hat{r} \rangle > 0$ so that $\hat{r}$ gets closer to r. This requires $\Delta R_H(\hat{r}) > 0$, which implies via Eq. (2) that $\Delta R_{nH}(\hat{r}) < 0$. So for $\hat{r} = r$ to be a stable fixed point, we require that the Hebbian rule slightly increase $\hat{r}$ and the non-Hebbian rule slightly decrease $\hat{r}$.

STDP and Frequency Dependence

The model curves in Figs 3, 4 are based on the plasticity rules described in Eqs (4) and (6). In the classical STDP experiments, the time delay Δt between presynaptic and postsynaptic spikes is varied, but the frequency of spike pairs, denoted by the period T is fixed. However in the frequency dependence experiments, the time between presynaptic and postsynaptic spikes is fixed, but the frequency of spike pairs is varied.

Experimental works do not report all necessary measurements for each neuron used in the experiments to fully constrain our model. Thus, we provide two comparisons to the data illustrating the frequency dependence of the model when subjected to the classic STDP protocol (see Fig. 4a). One comparison with all parameters identical for both pre before post and post before pre spike ordering (aside form $\hat{r}$ which is assumed to be constant for all neurons and frequencies and fitted for each ordering). Then, one comparison where parameters vary between orderings. The main point here is that the model captures the important characteristic of the data in both cases; that LTP is promoted as frequency increases. As expected the fits are better when more parameters are allowed to vary.

We assume for the sake of simplicity that the postsynaptic membrane potential V_i, and synaptic strength w_{ij} are constant.

We first consider the case when the presynaptic spike occurs before the postsynaptic spike. Equation (4) then becomes

$$\Delta w_{ij}^{H} = \mp\epsilon \frac{w_{ij}\left(V_i - V_{E/I}\right)e^{-\Delta t/\tau}}{C\hat{r}_j}, \tag{S.6}$$

and Eq. (6) becomes

$$\Delta w_{ij}^{nH} = \pm\epsilon \frac{w_{ij}\tau}{C}\left(V_i - V_{E/I}\right)\left(1 - e^{-T/\tau}\right), \tag{S.7}$$

since the time between postsynaptic spikes is T. For each pair of spikes these plasticity rules are each applied once, giving a total relative weight change after n spike pairs

$$\frac{\Delta w_{ij}}{w_{ij}} = \mp\epsilon n \frac{V_i - V_{E/I}}{C}\left(\frac{e^{-\Delta t/\tau}}{\hat{r}_j} - \tau\left(1 - e^{-T/\tau}\right)\right). \tag{S.8}$$

Only the Δw^{nH} rule is affected by a change in frequency, and it decreases as the frequency increases; meaning at high frequency the synapse will strengthen.

If the postsynaptic spike occurs before the presynaptic spike in a spike pair, the relevant Hebbian time delay is between a presynaptic spike, and the postsynaptic spike in the next pair of spikes. So Equation (4) then becomes

$$\Delta w_{ij}^{H} = \mp\epsilon \frac{w_{ij}\left(V_i - V_{E/I}\right)e^{-(T-\Delta t)/\tau}}{C\hat{r}_j}. \tag{S.9}$$

At low frequency Δw^{H} is small. Δw^{nH} is unchanged from above since it is not dependent on presynaptic activity.

Again, for each pair of spikes these plasticity rules are each applied once, giving a total relative weight change after n spike pairs

$$\frac{\Delta w_{ij}}{w_{ij}} = \mp\epsilon n \frac{V_i - V_{E/I}}{C}\left(\frac{e^{-(T-\Delta t)/\tau}}{\hat{r}_j} - \tau\left(1 - e^{-T/\tau}\right)\right). \tag{S.10}$$

In this case both the Δw^{H} and Δw^{nH} rules are affected by a change in frequency. As above, Δw^{nH} decreases as frequency increases, while Δw^{H} increases as frequency increases, producing a steeper curve than for pre before post ordering.

In the experiments stimulating rat CA1 and measuring the change in induced response (Fig. 4b), the temporal structure of firing activity was not recorded. This means we cannot construct a detailed model of expected synaptic weight change for our model. To compare qualitatively we assume the actual activity can be approximated by an STDP like spike pattern with pre before post spiking and we thus use Eqs. (S.8) and (S.10).

Spike Triplets

These derivations are similar to the standard pair STDP protocols, but include an additional presynaptic or postsynaptic spike. We start with post, Δt_1, pre, Δt_2, post triplet. In this case the Hebbian learning rule (Eq. (S.6)) is applied in response to the presynaptic spike and the second postsynaptic spike in the same triplet

$$\Delta w_{ij}^{H} = \mp\epsilon \frac{w_{ij}\left(V_i - V_{E/I}\right) e^{-\Delta t_2/\tau}}{C\hat{r}_j}. \tag{S.11}$$

There are two applications of the non-Hebbian learning rule (Eq. (S.7)), one for the time period between the two postsynaptic spikes within a triplet, and one for the time period between the last and first postsynaptic spikes in adjacent triplets

$$\Delta w_{ij}^{nH} = \pm\epsilon \frac{w_{ij}\tau}{C}\left(V_i - V_{E/I}\right)\left(2 - e^{-(\Delta t_1+\Delta t_2)/\tau} - e^{-(T-\Delta t_1-\Delta t_2)/\tau}\right), \tag{S.12}$$

where T is the period of the triplet stimulation. However, in the experiments we compare to in the main text, $T = 1$ s and the maximum value of $\Delta t_1 + \Delta t_2 = 20$ ms, so for τ around a few tens of milliseconds the last term is negligible and can be ignored, giving

$$\Delta w_{ij}^{nH} = \pm\epsilon \frac{w_{ij}\tau}{C}\left(V_i - V_{E/I}\right)\left(2 - e^{-(\Delta t_1+\Delta t_2)/\tau}\right), \tag{S.13}$$

Thus, the total relative weight change after n triplets is

$$\frac{\Delta w_{ij}}{w_{ij}} = \mp\epsilon n \frac{V_i - V_{E/I}}{C}\left(\frac{e^{-\Delta t_2/\tau}}{\hat{r}_j} - \tau\left(2 - e^{-(\Delta t_1+\Delta t_2)/\tau}\right)\right). \tag{S.14}$$

For the pre, Δt_1, post, Δt_2, pre triplet there are two applications of the Hebbian rule (Eq. (S.6), one for the first presynaptic spike in a triplet and the postsynaptic spike in that same triplet, and one for the second presynaptic spike in the triplet and the postsynaptic spike in the next triplet. Each of these potentially has a different $\hat{r}_j$ value.

$$\Delta w_{ij}^{H} = \mp\epsilon \frac{w_{ij}\left(V_i - V_{E/I}\right)}{C}\left(\frac{e^{-\Delta t_1/\tau}}{\hat{r}_j^a} + \frac{e^{-(T-\Delta t_2)/\tau}}{\hat{r}_j^b}\right). \tag{S.15}$$

As above, the second exponential term involving T is small and can be ignored

$$\Delta w_{ij}^{H} = \mp\epsilon \frac{w_{ij}\left(V_i - V_{E/I}\right)}{C\hat{r}_j} e^{-\Delta t_1/\tau}. \tag{S.16}$$

There is a single application of the non-Hebbian learning rule (Eq. (S.7)), for the time period between the two postsynaptic spikes in adjacent triplets

$$\Delta w_{ij}^{nH} = \pm\epsilon \frac{w_{ij}\tau}{C}\left(V_i - V_{E/I}\right)\left(1 - e^{-T/\tau}\right). \tag{S.17}$$

Again, the exponential term can be ignored

$$\Delta w_{ij}^{nH} = \pm\epsilon \frac{w_{ij}\tau}{C} \left(V_i - V_{E/I}\right). \tag{S.18}$$

Thus the total relative weight change after n triplets is

$$\frac{\Delta w_{ij}}{w_{ij}} = \mp\epsilon n \frac{\left(V_i - V_{E/I}\right)}{C} \left(\frac{e^{-\Delta t_1/\tau}}{\hat{r}_j} - \tau\right). \tag{S.19}$$

Tightly Balanced Currents

Equation (8) describes a stable state when currents produced by both the stimulus and the excitatory neurons are balanced by the inhibitory currents minus an amount corresponding to the number of spikes in the populations and the potential difference between the reset and threshold voltages. Therefore to configure the learning scheme to drive the network toward a balanced state, the $\gamma^{\alpha\beta}$ need to be made sufficiently small so that the stable state corresponds to excitatory currents that are much larger than needed to produce the spiking activity and therefore are balanced by almost equal inhibitory currents.

By considering the implementation of these learning rules in a network of firing rate neurons, the process of these plasticity rules driving a network toward an approximate balance between excitatory and inhibitory currents can be understood as a gradient descent.

We consider a network of neurons whose instantaneous firing rate $r_j(t)$ is given by the difference between its excitatory and inhibitory input currents which themselves correspond to a sum of firing rates of other neurons in the network, weighted by the synaptic strength between them

$$r_j(t) = \sum_{E \in \text{ExcitatoryPopulation}} W_{Ej} r_E(t) - \sum_{I \in \text{InhibitoryPopulation}} W_{Ij} r_I(t). \tag{S.20}$$

We can also construct $\hat{r}_j$ to be the product of synaptic strengths and firing rates

$$\hat{r}_j(t) = \sum_i W_{ij} r_i \tag{S.21}$$

We can construct a positive function E_j, corresponding to the error between the neuron's firing rate $r_j(t)$, and the estimated firing rate $\hat{r}_j(t)$

$$E_j(t) = \frac{1}{2}\left(r_j(t) - \hat{r}_j(t)\right)^2 \tag{S.22}$$

Taking a partial derivative with respect to a weight W_{ij} gives

$$\frac{\partial E(t)}{\partial W_{ij}} = -r_j(t) r_i(t) + \hat{r}_j(t) r_i(t). \tag{S.23}$$

Comparing Eq. (7) to Eq. (S.23) we can observe that in making a small gradient descent step, the first term corresponds to the R_H rule with presynaptic r_j

and postsynaptic r_i terms, and the second corresponds to the R_{nH} rule with a postsynaptic r_i term. The required relationship described by Eq. (2) is satisfied by the presence of $\hat{r}_j$ in the second term, noting that because the neurons no longer spike the Hebbian rule also becomes a differential and the Δt term in Eq. (2) is not required.

Thus the learning rules will attempt to minimise $E(t)$ and thus configure the network to a point at which $\hat{r}_j(t) \approx r_j(t)$. Then, if each of the excitatory and inhibitory currents are much larger than the firing rate, $I_{exc} = \sum_E W_{jE} r_E(t) >> r_j$, and $I_{inh} = \sum_I W_{jI} r_I(t) >> r_j$, then the incoming excitatory and inhibitory currents to neuron i are approximately balanced, $I_E \approx -I_I$.

In the context of our learning rules, the magnitude, and thus balance of the excitatory and inhibitory currents can be adjusted by tuning the $\gamma^{\alpha\beta}$ parameters to be sufficiently small.
